# IL-12-releasing nanoparticles for effective immunotherapy of metastatic ovarian cancer

**DOI:** 10.1038/s41563-025-02390-9

**Published:** 2025-10-31

**Authors:** Ivan S. Pires, Gil Covarrubias, Victoria F. Gomerdinger, Coralie Backlund, Eduardo Nombera Bueno, Margaret M. Billingsley, Mae Pryor, Apoorv Shanker, Ezra Gordon, Shengwei Wu, Andrew J. Pickering, Mariane B. Melo, Heikyung Suh, Darrell J. Irvine, Paula T. Hammond

**Affiliations:** 1 Koch Institute for Integrative Cancer Research, Massachusetts Institute of Technology, Cambridge, MA USA; 2 Department of Chemical Engineering, Massachusetts Institute of Technology, Cambridge, MA USA; 3 Department of Materials Science and Engineering, Massachusetts Institute of Technology, Cambridge, MA USA; 4 Department of Biological Engineering, Massachusetts Institute of Technology, Cambridge, MA USA; 5 Ragon Institute of Massachusetts General Hospital, Massachusetts Institute of Technology and Harvard University, Cambridge, MA USA; 6 Howard Hughes Medical Institute, Chevy Chase, MD USA; 7 Department of Immunology and Microbiology, Scripps Research Institute, La Jolla, CA USA; 8 Broad Institute of Massachusetts Institute of Technology and Harvard, Cambridge, MA USA

**Keywords:** Drug delivery, Cancer immunotherapy, Nanoparticles, Immunotherapy, Targeted therapies

## Abstract

Immunotherapies such as immune checkpoint inhibitors are effective in treating several advanced cancers, but these treatments have had limited success in metastatic ovarian cancer. Here we engineered liposomal nanoparticles carrying a poly-ʟ-arginine/poly-ʟ-glutamate coating that promotes their binding and retention on the surface of ovarian cancer cells. Covalent anchoring of the potent immunostimulatory cytokine interleukin-12 (IL-12) to phospholipid headgroups of the liposome core enabled the polymer-coated particles to concentrate IL-12 in disseminated ovarian cancer tumours following intraperitoneal administration. Shedding of the layer-by-layer coating and serum-protein-mediated extraction of IL-12-conjugated lipids from the liposomal core over time enabled IL-12 to disseminate in the tumour bed following rapid nanoparticle localization in tumour nodules. Optimized IL-12-polymer-coated nanoparticles promoted robust T cell accumulation in ascites and tumours in mouse models, extending survival compared with free IL-12 and sensitizing tumours to immune checkpoint inhibitors, eliciting strong immune responses and immune memory. Overall, these findings support the potential of these polymer-coated nanoparticles for the sustained delivery of IL-12 to disseminated metastatic ovarian cancer.

## Abstract (web summary)

Polymer-coated liposomes specifically target and deliver interleukin-12 to metastatic ovarian cancer, strongly sensitizing the tumours to combination therapy with immune checkpoint inhibitors.

## Main

Ovarian cancer (OC) treatment is particularly challenging due to its typically late diagnosis and metastatic spread^1,2^. A promising approach for late-stage cancer treatment is immunotherapy^3–5^. However, poor baseline lymphocyte infiltration and an immunosuppressive tumour microenvironment (TME) have correlated with limited benefits of immunotherapy in OC patients^6,7^. Immunostimulatory agents such as cytokines and costimulatory antibodies may have the potential to overcome these limitations, but the systemic administration of these therapeutics is severely constrained by dose-limiting toxicities^8,9^.

Nanoparticles (NPs) are promising vehicles to deliver immunotherapeutics^10,11^. In OC, NPs have been used to deliver various immunomodulatory agents such as nucleic acids^12,13^, proteins^14^ and small molecules^15,16^. We previously reported that NPs coated with poly-ʟ-arginine (PLR)/poly-ʟ-glutamate (PLE) bilayers via layer-by-layer (LbL) deposition showed selective binding to the surface of OC cells^17^. In mouse OC models, the administration of liposomal LbL NPs carrying the potent immunostimulatory cytokine interleukin-12 (IL-12) showed reduced toxicity over systemic IL-12 dosing but only modest therapeutic efficacy^18,19^. We hypothesized that the non-covalent nickel–histidine interaction used to tether IL-12 to these particles was very short-lived in vivo^20,21^, leading to the premature release of cytokine before uptake in tumours.

Here we demonstrate that the covalent conjugation of IL-12 to the liposomal core of LbL NPs greatly improves the targeting and retention of IL-12 in peritoneally disseminated OC tumours, enabling immunological and therapeutic effects not observed with free cytokine treatment, our prior rapid-release LbL NP chemistry or unlayered (UL) NPs. In part, these effects arise from the ability of N-aryl maleimide (Mal) LbL NPs to promote durable T cell infiltration and activation within tumours, thereby enhancing the magnitude and quality of tumour-specific T cell responses. When combined with immune checkpoint inhibitors (ICIs), a striking response and survival rate could be observed in immunologically ‘cold’ OC mouse models. Mechanistic investigations revealed that these LbL NPs rapidly accumulate in tumour nodules on intraperitoneal (i.p.) administration, shed the LbL coating and gradually release IL-12 lipid conjugates via lipid extraction mediated by serum proteins present in interstitial fluid. In particular, as neither UL nor layered LbL NPs lacking lipid release showed substantial benefit over free IL-12, both rapid LbL-mediated cancer cell targeting and slow cytokine release with sustained retention on cell membrane surfaces were crucial for therapeutic efficacy. These findings demonstrate the potential of ‘target-and-release’ NP designs to effectively concentrate cytokine in disseminated OC lesions and promote robust antitumour immunity.

## Dynamics of IL-12-conjugated LbL NPs in physiological fluids

**Fig. 1:**
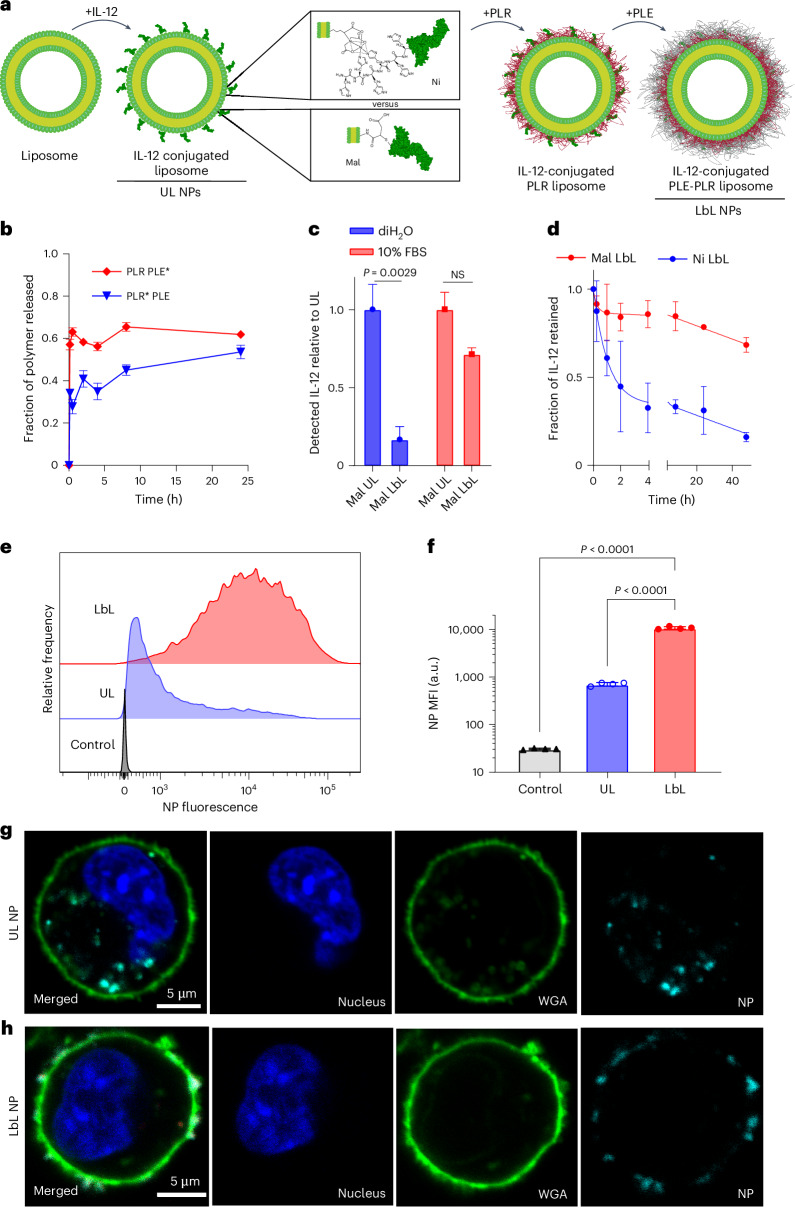
LbL NPs undergo dynamic reorganization on contact with physiological fluids. **a**, Schematic of LbL-NP assembly with either Mal or Ni linker chemistries for the conjugation of IL-12. **b**, Quantification of total PLE and PLR retained with LbL NPs following incubation in cell-free ascites fluid at 37 °C (* indicates a fluorophore-tagged polymer; *n* = 2–6 independent release assays; mean ± s.d.). **c**, Quantification of the total IL-12 available for monoclonal antibody binding from Mal NPs incubated in either diH_2_O or 10% FBS media (*n* = 2 independent batches with two technical replicates; mean ± s.d.). NS, not significant. **d**, Quantification of the total IL-12 released from LbL NPs on incubation in cell-free ascites fluid at 37 °C (*n* = 3–6 independent release assays; mean ± s.d.). **e**, Representative flow cytometry fluorescence histogram of HM-1 cells incubated with UL or PLE-coated LbL NPs for 4 h in vitro. **f**, Quantification of median fluorescence intensity (MFI) of treated HM-1 cells shown in **e** (*n* = 4 technical replicates; mean ± s.d.). **g**,**h**, Representative confocal images of HM-1 cells incubated with UL (**g**) or LbL (**h**) NPs for 4 h—UL images adjusted relative to LbL to visualize internalized NPs (blue, Hoechst 33342 nuclear stain; green, WGA cell surface stain; cyan, NPs). Data are representative of at least two independent experiments. Statistical comparisons in **c** and **f** were performed using two- and one-way analysis of variance (ANOVA), respectively, with Tukey’s multiple comparisons test. Source data

**Extended Data Fig. 1:**
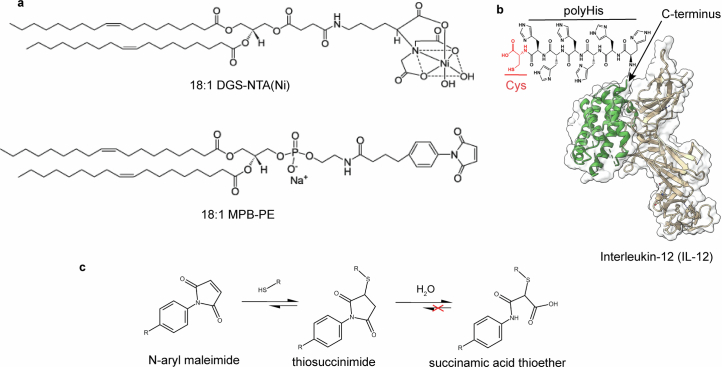
Headgroup-modified lipids for IL-12 conjugation. **a**, Chemical structure of headgroup-modified lipids with either chelated nickel or N-aryl maleimide. N-aryl maleimide was employed to prevent potential thiosuccinimide retro-Michael addition and subsequent thiol-exchange, as this headgroup favors thiosuccinimide hydrolysis into stable succinamic acid thioethers. **b**, IL-12 crystal structure derived from PDB 1F45 showing C-terminus used to engineered terminal poly-histidine (polyHis) tag and terminal cysteine. **c**, Reaction pathway for irreversible maleimide-based conjugation with thiols.

**Extended Data Fig. 2:**
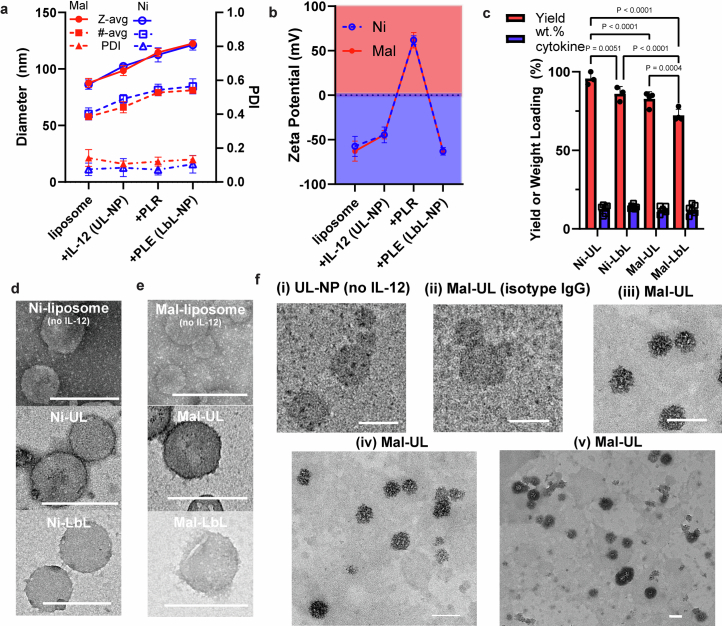
Synthesis of LbL-NPs conjugated with IL-12 via either maleimide-cysteine reaction or nickel-histidine interaction yields similar particle biophysical properties. **a**, Intensity-weighted hydrodynamic size (Z-avg), number average size (#-avg), and polydispersity index (PDI) of NPs during synthesis as measured via dynamic light scattering (mean ± s.d.). **b**, Zeta potential of NPs during synthesis as measured via electrophoretic mobility in deionized water (mean ± s.d.). **c**, Yield and weight loading of IL-12 for unlayered and layered particles with nickel-histidine linker (Ni-UL and Ni-LbL) and unlayered and layered particles with a maleimide-cysteine bond (Mal-UL and Mal-LbL) (mean ± s.e.m.). **d-e**, Negative-stain (NS) transmission electron microscopy (TEM) with phosphotungstic acid of particles during synthesis with nickel-containing lipids (**d**) and maleimide-containing lipids (**e**) - scale bars represent 200 nm. (f) Unlayered liposomal NPs devoid of IL-12 (UL-NP) or maleimide-cysteine IL-12 conjugated NPs (Mal-UL) were deposited on TEM copper grids followed by binding to either anti-IL-12 or isotype IgG antibody (Ab) and then exposed to 1 nm gold-labeled secondary anti-IgG FAbs. Shown are control UL-NPs without IL-12 conjugation exposed to anti-IL12 Ab (i), Mal-UL NPs incubated with isotype control IgG (ii) or Mal-UL NPs exposed to anti-IL-12 Ab (iii, iv, v). Scale bars 100 nm. Data are presented as mean values ± error with at least *n* = 3 independent batches of NPs. Statistical comparisons in **c** were performed using two-way analysis of variance (ANOVA) with Tukey’s multiple-comparisons test. Source Data

The overall design of the LbL NP system is shown in Fig. 1a. An immunostimulatory payload (here a single-chain form of the potent cytokine IL-12) is linked to the surface of a liposomal core particle, followed by sequential LbL deposition of a layer of positively charged PLR and then a layer of negatively charged PLE. To understand how the stability of the IL-12/NP association impacts the efficacy of this system, we compared the previously used non-covalent nickel–polyhistidine (Ni) interaction^19^ with a new formulation: covalently bonding Mal groups on NP lipids to free cysteine residues of IL-12 (ref. ^22^; Supplementary Table 1 and Extended Data Fig. 1a–c). IL-12-conjugated NPs were synthesized with both linker chemistries (Ni or Mal) in either UL or PLR/PLE-layered (LbL) formats. Ni and Mal NPs had similar sizes (Extended Data Fig. 2a), zeta potentials (Extended Data Fig. 2b), yields (>70%) and loadings of IL-12 (~10–13 wt%, corresponding to ~50 IL-12 molecules per particle^19^ (Extended Data Fig. 2c–e)). Immunogold staining to detect the cytokine revealed a homogeneous coverage of UL or LbL NPs with IL-12 (Extended Data Fig. 2f).

LbL NPs of this type are stored in deionized water (diH_2_O) to maintain colloidal stability and diluted in 5% dextrose for isotonic in vivo use^17,18,23^. However, exposure to the high ionic strength of i.p. fluid induces a partial rearrangement of the LbL film. Incubation in tumour-bearing ascites fluid from the highly metastatic OC cell line OV2944-HM-1 (HM-1 (ref. ^24^)) caused a burst release of ~40% of the PLR and ~60% of the PLE polymer, stabilizing thereafter (Fig. 1b). Similar release occurred in buffered saline, indicating that ionic strength drives polymer shedding (Supplementary Fig. 1a).

Given the LbL film reorganization on contact with physiological fluids, we assessed particle-bound IL-12 accessibility by capturing Mal NPs on microtitre plates and measuring the binding of an IL-12-specific antibody via enzyme-linked immunoassay (ELISA; Supplementary Fig. 1b). LbL coating reduced IL-12 accessibility by ~90% in diH_2_O, but exposure to a serum-containing buffer restored most antibody binding (Fig. 1c). Both UL and LbL NPs induced similar IL-12 signalling in reporter cells with serum (Supplementary Fig. 1c,d). IL-12 exposure enables signalling and potential release from particles, either by Ni/His-tag disruption (Ni particles) or protein-mediated extraction of lipid-anchored IL-12 (Mal particles). The incubation of fluorescent IL-12-tagged LbL NPs in ascites fluid showed >50% IL-12 release from Ni LbL particles in 2 h, whereas Mal LbL particles retained ~70% at 48 h (Fig. 1d). Finally, LbL NPs retained effective cancer cell targeting in physiological conditions. PLE-coated NPs have shown selective binding to OC versus healthy cells^17,25,26^. When incubated with HM-1 cells in complete media, LbL-coated NPs showed more than tenfold higher OC cell association than uncoated particles (Fig. 1e,f). Confocal imaging confirmed Mal LbL NPs localized mainly on the cell membrane 4 h post-dosing, whereas Mal UL NPs were internalized, consistent with prior Ni-IL-12 NP studies. This indicates that despite partial polymer shedding, the remaining LbL film effectively mediates cancer cell surface targeting (Fig. 1g,h).

Altogether, these results indicate rapid LbL coating reorganization in vivo, exposing IL-12 to promote immune engagement and maintaining effective OC cell targeting. The Ni:His-tag linkage enables rapid IL-12 release, whereas covalently anchored IL-12 shows much slower, sustained release.

## Tumour binding and prolonged IL-12 retention by LbL NPs

**Extended Data Fig. 3:**
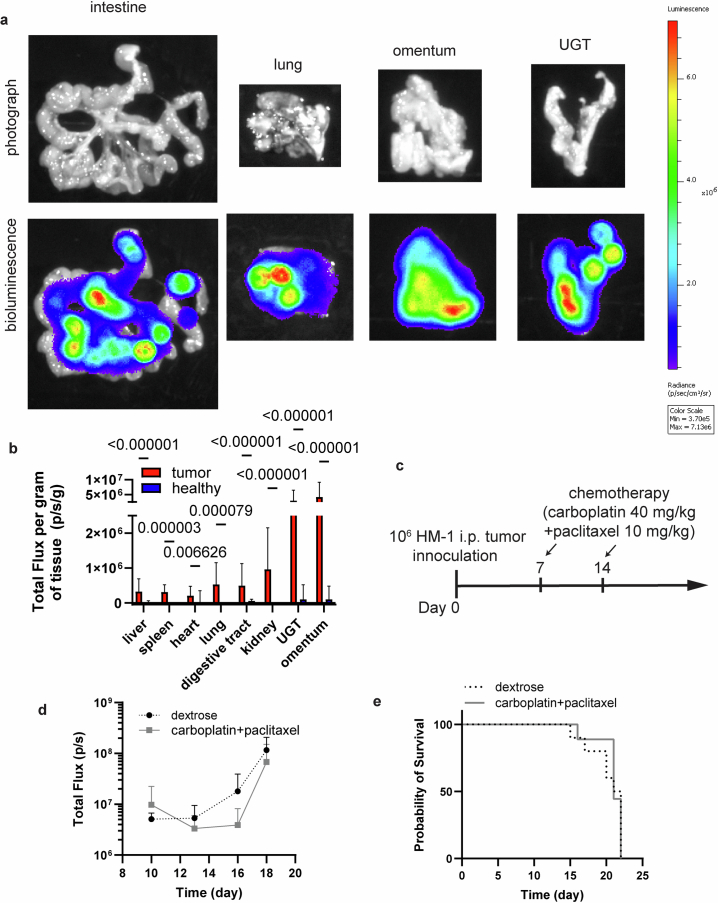
Characterization of metastatic ovarian cancer model - OV2944-HM1. **a-b**, B6C3F1 mice were inoculated with 10^6^ HM-1-luc tumor cells i.p. on day 0 (*n* = 40 animals) or left as naïve healthy animals (*n* = 10 animals). Shown are representative, intestine, lung, omentum and UGT ex-vivo IVIS BLI images (**a**) and quantitation of BLI signal in healthy organs compared to organs two weeks after tumor inoculation (mean ± s.d., **b**). Statistical comparisons in **b** were performed using the nonparametric Mean-Whitney test with correction for multiple comparisons based on a false discovery rate of 1%. **(c-e)** B6C3F1 mice were inoculated with 10^6^ HM-1-luc tumor cells i.p. on day 0 and either treated i.p. with dextrose (*n* = 10 animals) or carboplatin (40 mg/kg) and paclitaxel (10 mg/kg, *n* = 9 animals) on days 7 and day 14. Shown are the experimental timeline **(c)**, in vivo IVIS whole-animal i.p. BLI readings (mean ± s.d., **d)**, and overall survival **(e)**. Source Data

We next characterized the in vivo pharmacokinetics of IL-12 delivery via LbL NPs in a model of disseminated OC^24^. Seven days after an i.p. injection of luciferase-expressing HM-1 cells (HM-1-luc), tumour nodules appear mainly in the intestines, omentum and urogenital tract (UGT), with a preference for omentum and UGT, mirroring human metastatic OC (Extended Data Fig. 3a,b)^27^. Tumour cells are also detected at lower levels in thoracic tissues (lungs and heart), indicating spread beyond the i.p. space (Extended Data Fig. 3a,b). The HM-1 model is highly resistant to platinum-based therapies, alone^28^ or combined with dose-dense weekly paclitaxel^29^, reflecting chemoresistant metastatic OC (Extended Data Fig. 3c–e).

**Fig. 2:**
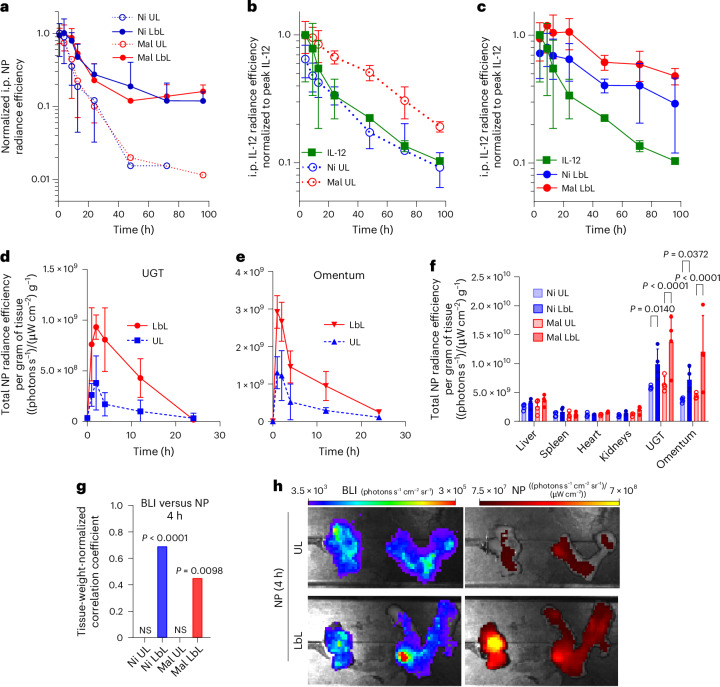
LbL coating enables targeting of tumour tissue in vivo and enhanced i.p. retention of NPs and IL-12. **a**–**c**, B6C3F1 mice (*n* = 8 animals per group for 0–24 h and *n* = 3 animals per group for 24–96 h) inoculated with 10^6^ HM-1-luc tumour cells on day 0 were administered fluorescently tagged NPs carrying 20 µg of IL-12 (or an equivalent dose of free IL-12) on day 14. Whole-animal imaging NP fluorescence (**a**) and IL-12 fluorescence for UL (**b**) and LbL (**c**) NP treatments are shown from the i.p. space collected over time post-dosing (mean ± s.d.). **d**,**e**, B6C3F1 mice (*n* = 4 animals per group) inoculated with 10^6^ HM-1-luc tumour cells on day 0 were administered 130 µg of lipids in fluorescently tagged versions of LbL NPs or UL NPs (devoid of IL-12) on day 14. UGT and omentum tissues were harvested at 1, 2, 4, 12 and 24 h after dosing and imaged ex vivo via IVIS. Weight-normalized tissue NP fluorescence in UGT (**d**) and omentum (**e**) are shown (mean ± s.d.). **f**–**h**, B6C3F1 mice (*n* = 4 animals per group) were treated as that in **a**–**c** (**f**–**h**, respectively). Four hours after dosing, animals were euthanized, and tissues were analysed ex vivo via IVIS. Weight-normalized tissue NP fluorescence (mean ± s.d.; **f**) are shown, along with Pearson’s correlation coefficient for groups (eight tissues with four replicates per group) with significant (*P* < 0.05) correlation between weight-normalized tissue NP fluorescence and BLI 4 h after dosing (**g**), and representative omentum and UGT tissue IVIS BLI and NP fluorescence images for LbL NPs and UL NPs (**h**). Statistics derived from using all *n* from experiment with each animal as a data point. For **g**, the correlation significance is performed based on a two-sided *t*-test analysis with the null hypothesis of no (*r* = 0) correlation and no adjustments for multiple comparisons. Group statistical comparisons in **f** were performed using a two-way ANOVA with Tukey’s multiple comparisons test. Source data

**Extended Data Fig. 4:**
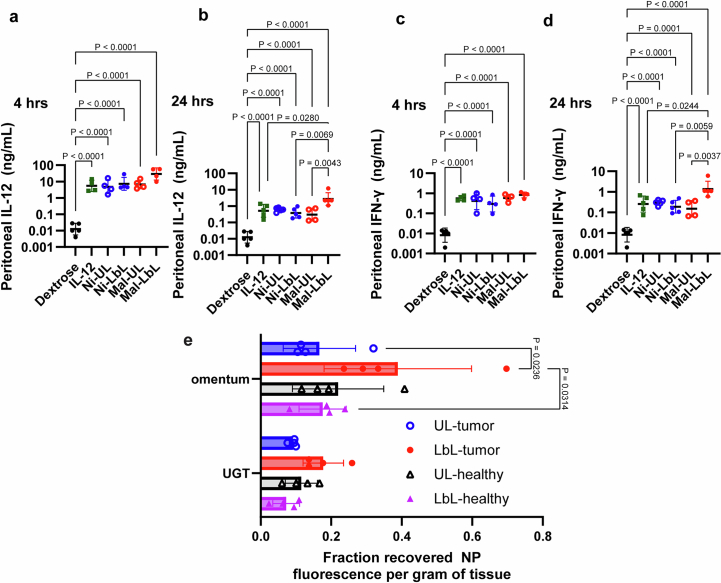
Mal-LbL NPs localize immune response to peritoneum and effectively target cancer tissue in i.p. space. **a-d**, B6C3F1 mice (*n* = 4-5 animals/group/timepoint) inoculated with 10^6^ HM-1-luc tumor cells on day 0 were administered NPs carrying 20 µg IL-12 (or an equivalent dose of free IL-12) on day 14. Four or 24 hours after dosing, mice were euthanized and ascites was extracted with a syringe. Shown are the quantification of ascites IL-12 concentration four (**a**, mean ± s.d.) and 24 hrs (**b**, mean ± s.d.) after dosing, and quantification of IFN-γ four (**c**, mean ± s.d.) and 24 hrs after dosing (**d**, mean ± s.d.). **e**, Both healthy B6C3F1 (n = 4/group) and B6C3F1 mice (n = 4/group) inoculated with 10^6^ HM-1-luc tumor cells on day 0 were administered 20 µg fluorescently-tagged LbL-NPs or UL-NPs on day 14. Tissue was harvested at 4 hrs after dosing and imaged ex-vivo via IVIS. Shown are weight-normalized recovered NP fluorescence (**c**, mean ± s.d.). Each data point represents one animal in the experiment. Source Data

Using fluorescently tagged IL-12 and lipids, we tracked NP and IL-12 clearance from the i.p. space via whole-animal imaging. UL NPs showed exponential NP signal decay over time, whereas LbL NPs cleared partially over 24 h, with ~10% retained from 1 to 4 days (Fig. 2a). IL-12 clearance was impacted by both LbL coating and linkage chemistry: IL-12 on Ni UL NPs cleared rapidly, indistinguishable from free IL-12 (Fig. 2b). LbL coating of Ni NPs prolonged IL-12 persistence, with Mal UL NPs showing similar cytokine kinetics (Fig. 2b,c). Cytokine delivered by Mal LbL NPs cleared most slowly, with ~50% IL-12 signal retained at 4 days (Fig. 2c). In particular, the clearance of IL-12 was slower than the lipid carrier, suggesting the dissociation of cytokine from Mal LbL NPs. The analysis of ascites fluid confirmed the enhanced retention of IL-12 (Extended Data Fig. 4a,b) and increased local immune stimulation, evidenced by higher interferon-gamma (IFN-γ) levels (Extended Data Fig. 4c,d).

To define NP accumulation kinetics in tumours, fluorescent UL or LbL NPs (without IL-12) were injected via i.p. administration into HM-1-luc-tumour-bearing mice. Ex vivo imaging of tumour tissues showed that LbL NPs rapidly associated with high-tumour-burden sites (Fig. 2d,e), peaking at ~1 h in the UGT and ~2 h in the omentum, with twofold–fivefold higher fluorescence than UL NPs. Evaluating Ni or Mal NPs distribution at 4 h post-injection showed low uptake in other organs (Fig. 2f) and the increase in LbL NP association with the high-tumour-burdened tissue (that is, omentum) was only observed in tumour-bearing animals (Extended Data Fig. 4e). Indeed, only LbL NPs exhibited a significant correlation between tumour bioluminescence intensity (BLI) and NP fluorescence (Fig. 2g,h and Supplementary Fig. 2), consistent with the LbL NP targeting of the disseminated tumours.

**Fig. 3:**
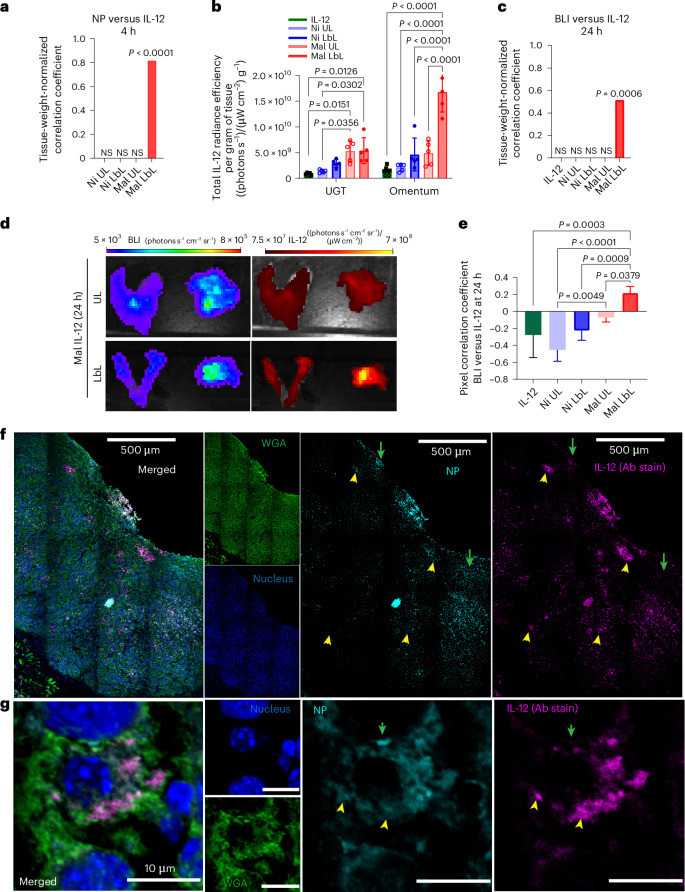
Mal LbL NPs efficiently target and deliver IL-12 to OC tumour nodules. **a**–**e**, B6C3F1 mice (*n* = 4–5 animals per group) inoculated with 10^6^ HM-1-luc tumour cells on day 0 were administered fluorescently tagged NPs carrying 20 µg of IL-12 on day 14. Four hours or 1 day after dosing, the animals were euthanized, and tissues were analysed ex vivo via IVIS. Pearson’s correlation coefficient is shown for groups (eight tissues with five replicates per group) with significant (*P* < 0.05) correlation between weight-normalized tissue NP fluorescence and IL-12 fluorescence 4 h after dosing (**a**), weight-normalized tissue IL-12 fluorescence 1 day after dosing in UGT and omentum (mean ± s.d.; **b**), Pearson’s correlation coefficient for groups (eight tissues with five replicates per group) with significant (*P* < 0.05) correlation between weight-normalized tissue IL-12 fluorescence and BLI 1 day after dosing (**c**), representative omentum and UGT tissue IVIS BLI and IL-12 fluorescence images for Mal UL and Mal LbL (**d**) and pixel-by-pixel Spearman’s correlation coefficient between IL-12 fluorescence and BLI 1 day after dosing from IVIS images across all tissues (**e**; mean ± s.d., eight tissues with five replicates per group). **f**,**g**, B6C3F1 mice were treated as that in **a**. One day after dosing, Mal LbL NP animals were euthanized, and the omentum containing tumour nodules was frozen in OCT compound and then frozen, sectioned and stained for confocal microscopy analysis. Representative confocal microscopy images of tumour nodules in omental tissue are shown at low (**f**) and high (**g**) magnifications. The green arrows indicate areas with a high NP signal relative to IL-12, whereas yellow arrowheads indicate areas with high IL-12 relative to NPs. Statistics derived using all *n* from the experiment with each animal as a data point. For **a** and **c**, correlation significance performed based on a two-sided *t*-test analysis with the null hypothesis of no (*r* = 0) correlation and no adjustments for multiple comparisons. Group statistical comparisons were performed using two-way ANOVA for **b** and one-way ANOVA for **e** with Tukey’s multiple comparisons test. Source data

We examined IL-12 distribution in OC tissues and its spatial relation to the NP carriers. At peak NP accumulation (~4 h), spatial correlation between NP and IL-12 signals was only significant for Mal LbL NPs (Fig. 3a and Supplementary Fig. 2), indicating that neither the LbL film of Ni LbL nor the covalent conjugation of Mal UL alone enabled the retention of cytokine on the particle carrier over this time course in vivo. At 24 h, Mal LbL NPs increased IL-12 retention in tumour-bearing UGT and omentum tissues by five- and tenfold versus free cytokine, respectively (Fig. 3b). Importantly, IL-12 fluorescence from Mal LbL NPs significantly correlated with the tumour BLI, demonstrating enhanced targeting and retention compared with UL or Ni NPs (Fig. 3c and Supplementary Fig. 2). Moreover, ex vivo pixel-by-pixel in vivo imaging system (IVIS) analysis revealed a positive correlation between tumour BLI and IL-12 fluorescence only for Mal LbL NP delivery, indicating improved tumour targeting and retention (Fig. 3d,e). This correlation was also observed within the omentum tissue, confirming that the association with targeted tissues was not random (Supplementary Fig. 3a,b).

Histological analysis of omental tumour nodules 24 h after dosing revealed that the Mal LbL NPs could efficiently penetrate the tumour tissue and disseminate IL-12 (Fig. 3f). Consistent with the expected lipid-IL-12-conjugate release from Mal LbL and the fact that the NP and IL-12 signals were mostly correlated across the tissue, we could observe regions with only IL-12 or only NP signal. Furthermore, at high magnification, we could observe that the Mal LbL NPs appeared to diffusely stain the membrane of cells in the tumour with patches of high IL-12 or high NP signal (Fig. 3g). These segregating signals suggest the release of IL-12 from the NP cores over time, on a timescale substantially slower than the time required for the NPs to effectively localize to tumour nodules.

## Tumour-targeted IL-12 delivery boosts therapy without toxicity

**Fig. 4:**
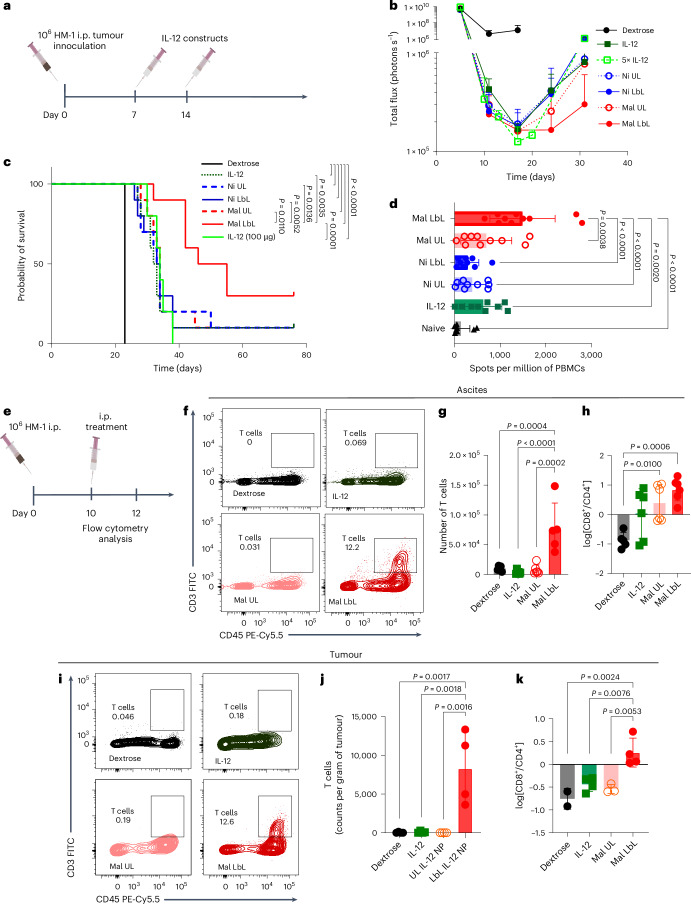
Mal LbL NP drives strong antitumour activity and boosts T cell tumour infiltration. **a**–**d**, B6C3F1 mice (one cohort of *n* = 10 animals per group) inoculated with 10^6^ HM-1-luc tumour cells on day 0 were treated on days 7 and 14 with 20 µg of IL-12 as a free cytokine or conjugated to NPs. Experimental timeline (**a**), IVIS whole-animal i.p. BLI readings (mean ± s.d.; **b**) and overall survival (**c**). On day 30, PBMCs of surviving and naive mice (*n* = 5 animals) were analysed via IFN-γ ELISpot restimulated with HM-1-luc tumour cells. Quantitation of spots detected (mean ± s.d.; **d**) are shown. **e**–**k**, B6C3F1 mice inoculated with 10^6^ HM-1 tumour cells on day 0 were treated on day 10 with 20 µg of IL-12 as a free cytokine or conjugated to Mal NPs (UL and LbL). Two days after dosing, ascites (*n* = 6 per group) and i.p. tumour nodules (primarily omentum tissue, *n* = 4 per group) were harvested and processed for flow cytometry analysis. Timeline for experiment (**e**), representative flow plots of T cell (CD45^+^CD3^+^) in ascites fluid (**f**), quantitation of T cells in ascites fluid (**g**), quantitation of CD8^+^ to CD4^+^ T cell ratio in ascites fluid (**h**), representative flow plots of T cell (CD45^+^CD3^+^) in tumour nodules (**i**), quantitation of T cells in tumour nodules (**j**) and quantitation of CD8^+^ to CD4^+^ T cell ratio in tumour nodules (**k**). Statistics derived using all *n* from experiment with each animal as a data point. *P* values were determined by the log-rank (Mantel–Cox) test (**c**) and one-way ANOVA followed by Tukey’s multiple comparison test (**d**, **g**, **h**, **j** and **k**). Source data

To assess the therapeutic impact of enhanced IL-12 targeting to ovarian tumours achieved by Mal LbL NPs, HM-1-luc-tumour-bearing mice were treated with 20 µg of IL-12 administered as free cytokine or NP formulations on days 7 and 14, or a five times higher dose of the free cytokine (to determine whether a higher dosing of free cytokine could compensate for its rapid clearance; Fig. 4a). Three days following the first dose, a dramatic drop in tumour BLI was observed for all IL-12 treatments (Fig. 4b). However, except for the Mal-LbL-NP-treated group, tumour signals began to rebound by day 24, ultimately leading to similar increases in median survival of ~33 days, compared with 23 days for the untreated tumours (Fig. 4c). By contrast, Mal LbL NPs delayed tumour recurrence, increasing the median survival to 44 days, with ~30% of animals achieving complete tumour clearance. IFN-γ ELISpot analysis of peripheral blood lymphocytes on day 30 revealed a stronger tumour-specific T cell response induced by Mal LbL NP treatment compared with all other groups (Fig. 4d). When mice that rejected their tumours were rechallenged with i.p. administration of fresh tumour cells on day 100, all animals showed rapid tumour clearance (Supplementary Fig. 4a) and survived, whereas all naive mice succumbed to the tumour challenge (Supplementary Fig. 4b), indicating a successful development of protective antitumour memory.

**Extended Data Fig. 5:**
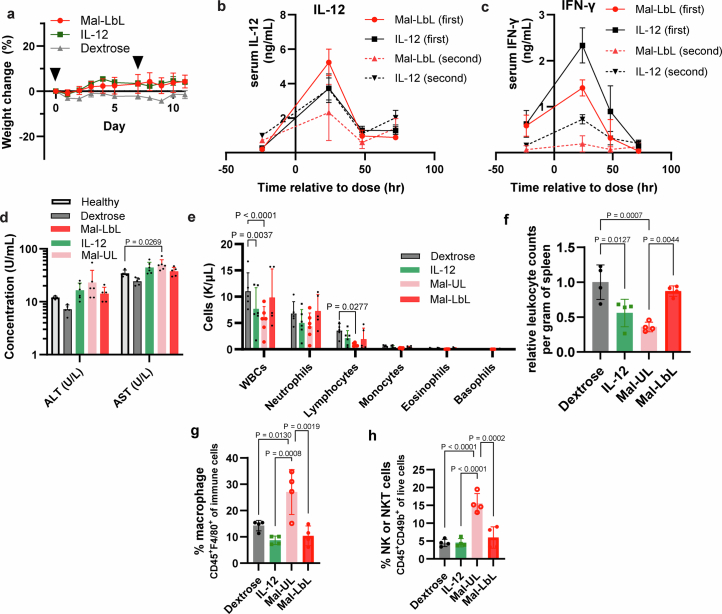
Intraperitoneal dosing of 20 µg of Mal-LbL NPs does not cause systemic toxicity. **a**, healthy B6C3F1 mice were treated on days 0 and 7 with 20 µg of IL-12 as a free cytokine or conjugated to Mal LbL-NPs. Shown are the percentage change of body weight. **b-c**, B6C3F1 mice (1 cohort *n* = 5 animals/group) inoculated with 10^6^ HM-1-luc tumor cells on day 0 were treated on days 7 and 14 with 20 µg of IL-12 as a free cytokine or conjugated to Mal-LbL NPs. Serum was collected from mice one day before dosing as well as 24, 48 and 72 hrs after dosing. Shown are quantitation of serum IL-12 (**b**) and serum IFN-γ (**c**). **d-h**, B6C3F1 mice inoculated with 10^6^ HM-1 tumor cells on day 0 were treated on days 10 with 20 µg of IL-12 as a free cytokine or conjugated to Mal NPs (UL and LbL). Two days after dosing blood (n = 5-6/group) and spleens (n = 4/group) were harvested and sent for a complete blood panel or processed for flow cytometry analysis, respectively. Shown are serum levels of liver damage markers (alanine transaminase – ALT - and aspartate aminotransferase - AST) compared to healthy mice controls (*n* = 4, **d**), complete blood count panel (**e**), quantitation of live leukocyte (CD45^+^) counts in spleen (**f**), and percentage of macrophage (**g**), NK (**h**) cell in splenocytes. Statistical comparisons performed using two-way (**d, e**) or one-way (**f, g, h**) analysis of variance (ANOVA) with Tukey’s multiple-comparisons (liver enzyme measurement was compared to healthy controls). In all panels, each point corresponds to a single animal, with values shown as mean ± standard deviation. Source Data

We next sought to further determine the safety of this treatment. Healthy mice received two 20 µg doses of free IL-12 or Mal LbL NPs 1 week apart. Both treatments were well tolerated, with stable body weight (Extended Data Fig. 5a). Peak exposures of IL-12 in the blood at 24 h post-dosing represent <0.1% of the administered dose (Extended Data Fig. 5b). Serum IFN-γ levels, reflecting IL-12 activity, were almost twofold lower in Mal LbL versus free IL-12 1 day after dosing (Extended Data Fig. 5c), despite higher i.p. IFN-γ (Extended Data Fig. 4d). The second dose of IL-12 elicited a blunted IFN-γ response, consistent with prior IL-12 therapy reports^30^, and levels returned to baseline within 2–3 days after dosing in both treatment groups.

To further evaluate toxicity, mice bearing 10-day HM-1 i.p. tumours were treated with dextrose, free IL-12 or IL-12 conjugated to Mal UL or Mal LbL NPs. Two days post-dosing, blood and spleens were analysed (Fig. 4e). In addition to IFN-γ signalling, systemic IL-12 administration is known to increase markers of liver toxicity, induce transient cytopenia and alter the splenic immune cell profile^31–37^. Although none of the treatments caused major liver damage as measured by levels of liver enzymes in the bloodstream, Mal UL increased levels compared with healthy controls (Extended Data Fig. 5d). Both free IL-12 and Mal UL reduced white blood cell counts compared with vehicle control mice, which was not observed with Mal LbL treatment (Extended Data Fig. 5e). Untargeted IL-12 treatments also induced reduced leucocyte counts in the spleen and Mal UL NPs elevated the levels of splenic macrophage and natural killer (NK) cells (Extended Data Fig. 5f–h), consistent with the expected effects of systemic IL-12 exposure. Histopathological evaluation of haematoxylin and eosin (H&E)-stained sections of the liver, uterus, ovaries, intestines, kidneys and spleen on day 2 post-treatment showed no direct organ toxicity. Sheets of neoplastic cells were abutting most organs and occasionally invading the liver capsule and encroaching into the liver parenchyma in all groups including the dextrose group. However, mostly, neoplastic cells were present on capsular surfaces or embedded within peritoneal adipose tissue (omentum and/or mesentery). There were no major differences between various IL-12 treatment groups compared with the dextrose-treated group, suggesting that the IL-12 constructs were not associated with direct tissue toxicity in this acute study.

**Extended Data Fig. 6:**
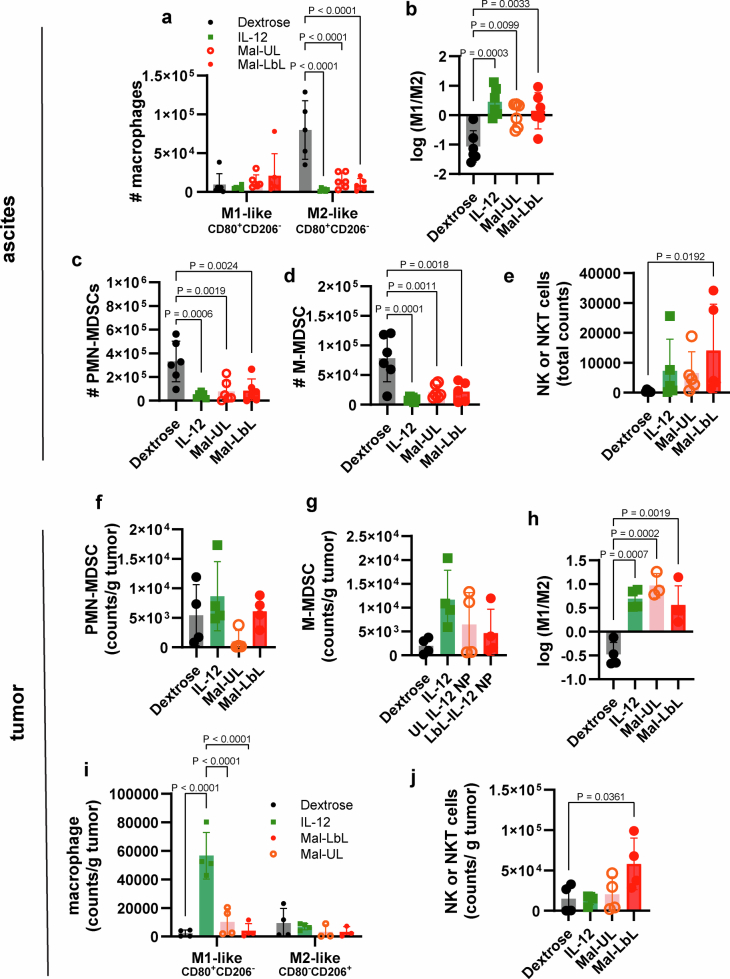
Immune phenotyping of cells in ascites fluid and tumor tissues. **a-j**, B6C3F1 mice inoculated with 10^6^ HM-1 tumor cells on day 0 were treated on days 10 with 20 µg of IL-12 as a free cytokine or conjugated to Mal NPs (UL and LbL). Two days after dosing ascites (*n* = 6 animals/group) and i.p. tumor nodules (primarly omentum tissue, *n* = 4 animals/group) were harvested and processed for flow cytometry analysis. Shown are total counts of M1-like and M2-like macrophages **(a)**, logarithmic of M1-like to M2-like macrophages ratio (**b**), and total counts of PMN-MDSC (**c**), M-MDSC (**d)**, and NK cells (**e**) in ascites fluid. Also shown are total counts of PMN-MDSC (**f**) and M-MDSC (**g**), logarithmic of M1-like to M2-like macrophages ratio (**h**), and total counts of M1-like and M2-like macrophages (**i**), and NK cells (**j**) in tumor nodules. Statistical comparisons were performed using two-way (**a, i**) or one-way (**b, c, d, e, h, j**) analysis of variance (ANOVA) with Tukey’s multiple comparisons (liver enzyme measurement was compared to healthy controls). In all panels, each point corresponds to a single animal, with values shown as mean ± standard deviation. Source Data

To examine the effects of IL-12 therapy on the immune response in the local tissue, we next analysed leucocytes in the ascites (peritoneal fluid) and tumour nodules via flow cytometry 2 days following a single dose of free IL-12 or IL-12 NPs on day 10 post-HM-1 inoculation (Fig. 4e). Within the ascites, all IL-12 treatments depleted protumourigenic CD206^+^CD80^−^ (M2-like) macrophages (Extended Data Fig. 6a), shifting the macrophage population towards a predominantly tumouricidal CD206^−^CD80^+^ M1-like phenotype (Extended Data Fig. 6b). Polymorphonuclear and monocyte-related myeloid-derived suppressor cells in the ascites, which can hinder the development of an effective immune response^38^, were also reduced for all IL-12 treatment groups (Extended Data Fig. 6c,d), whereas levels of NK cells increased (Extended Data Fig. 6e). However, only Mal LbL treatment substantially increased T cell accumulation in the ascites fluid (Fig. 4f,g). Characterization of the T cell subtypes revealed a shift towards an increased CD8:CD4 ratio (Fig. 4h), which is associated with improved outcomes in human OC patients^39^.

In the extracted tumour nodules, IL-12 treatments did not cause major effects in either polymorphonuclear myeloid-derived suppressor cells (Extended Data Fig. 6f) or M-myeloid-derived suppressor cell levels (Extended Data Fig. 6g). All IL-12-based treatments polarized the macrophage population from a predominantly M2-like phenotype towards a predominantly M1-like phenotype (Extended Data Fig. 6h). However, only free IL-12 treatment led to a substantial increase in the number of M1-like tumouricidal macrophages (Extended Data Fig. 6i), suggesting a bias towards monocyte-driven immune response from systemic IL-12 treatment. On the other hand, Mal LbL NPs increased NK cell infiltration (Extended Data Fig. 6j). Moreover, Mal LbL NP treatment triggered a dramatic ~50-fold increase in T cell infiltration in tumour nodules and increase in the ratio of CD8^+^ to CD4^+^ T cells, which was not observed for free IL-12 or UL particles (Fig. 4i–k). These results highlight that the specific delivery of the cytokine to cancer cells, together with its sustained presence in the TME, is essential for driving lymphocyte infiltration—an outcome not observed with free cytokine administration. This enhanced infiltration, together with elevated IFN-γ levels detected in the ascites following Mal LbL treatment, probably contributes to the generation of stronger tumour-specific T cell responses, as observed in the ELISpot assays. Sustained local cytokine activity may, therefore, both expand and potentiate the cytotoxic T cell pool capable of recognizing and eliminating tumour cells.

## IL-12 delivery is regulated by liposomal membrane composition

**Fig. 5:**
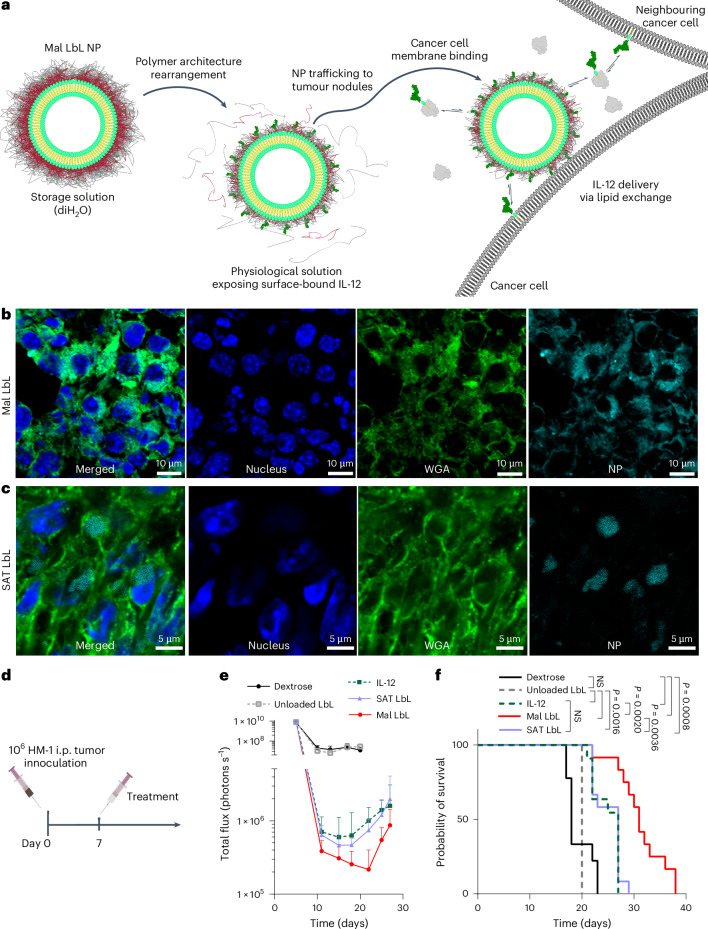
Mal LbL NP efficacy depends on lipid-exchange-driven distribution within tumour nodules. **a**, Schematic of the proposed mechanism of tumour-targeted IL-12 lipid conjugate dissemination from Mal LbL NPs. **b**,**c**, B6C3F1 mice inoculated with 10^6^ HM-1-luc tumour cells on day 0 were administered fluorescently tagged Mal LbL of SAT LbL NPs carrying 20 µg of IL-12 on day 14. One day after dosing, the animals were euthanized, and the omentum containing tumour nodules was frozen in an OCT compound and then frozen, sectioned and stained for confocal microscopy analysis. Representative high-magnification confocal images of omental tumour nodules from Mal LbL (**b**) and SAT LbL (**c**). **d**–**f**, B6C3F1 mice (two cohorts for *n* = 7 and 5 animals per group per cohort) inoculated with 10^6^ HM-1-luc tumour cells on day 0 were treated on day 7 with NP vehicle control (unloaded LbL), 20 µg of IL-12 as a free cytokine or conjugated to Mal LbL or SAT LbL. Experimental timeline (**d**), one representative in vivo IVIS whole-animal i.p. BLI readings from two independent experiments (mean ± s.d., *n* = 7 animals per group; **e**) and overall survival (**f**). Statistics derived using all *n* from experiment with each animal as a data point. Statistical comparisons between survival curves were performed using a log-rank (Mantel–Cox) test. Source data

**Extended Data Fig. 7:**
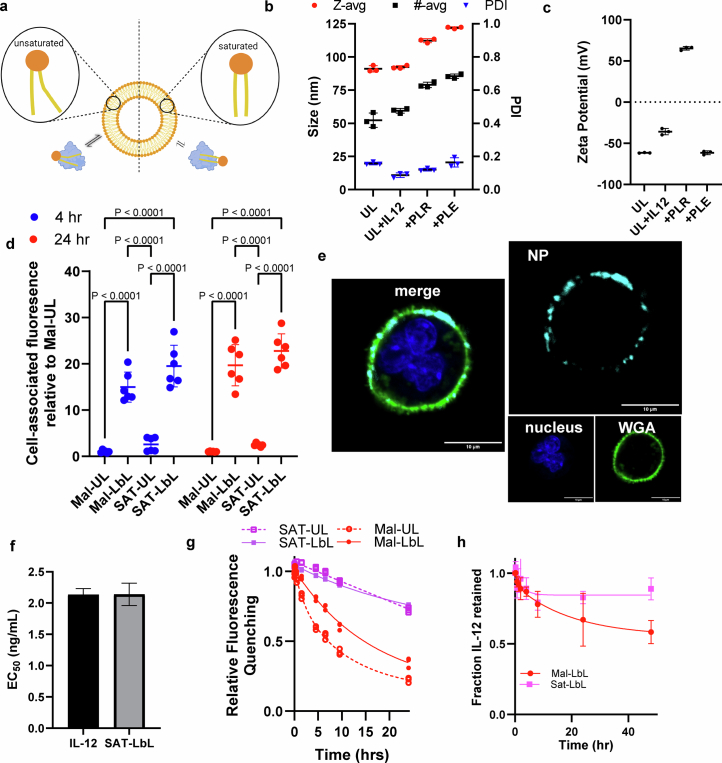
Characterization of Mal IL-12 NPs composed of saturated (SAT) lipids. **a**, Illustration of liposome bilayer composition effect on lipid exchange rate with serum proteins. **b**, Intensity-weighted hydrodynamic size (Z-avg), number average size (#-avg), and PDI of NPs during synthesis as measured via DLS (*n* = *3* technical replicates from representative batch, mean ± s.d.). **c**, Zeta potential of NPs during synthesis as measured via electrophoretic mobility in deionized water (*n* = *3* technical replicates from representative batch, mean ± s.d.). **d**, Association of NP fluorescence with HM1 cells *in vitro* relative to unlayered NPs after 4 and 24 hours of incubation (*n* = *6* replicates from two independent batches, mean ± s.d.). **e**, Representative confocal microscopy images of HM-1 cells dosed with SAT-LbL for 4 hours. **f**, Calculated IL-12 EC_50_ of IL-12 compared to SAT-LbL NPs from HEK-Blue IL-12 assay (mean ± s.e.m.). **g**, Assessment of de-quenching from fluorophore detachment from unsat and SAT NPs when incubated with 100% FBS at 37 °C – curves represent the best fit of a two-phase decay model. **h**, Quantification of IL-12 retention with Mal-LbL or SAT-LbL upon incubation with 100% FBS (*n* = *4-9* independent release experiments from two batches, mean ± s.d.) – curves represent the best fit of a two-phase decay model. Statistical comparisons performed in **d** using two-way analysis of variance (ANOVA) with Tukey’s multiple-comparisons. Data are representative of at least two independent experiments. Panel **a** created with BioRender.com. Source Data

Membrane lipids in vivo can be extracted from bilayers by albumin and other serum components and undergo constant exchange with serum lipids^40–43^. We hypothesized that the gradual release of IL-12 lipid conjugates from Mal LbL NPs through this process was important for optimal cytokine activity, as this could promote the dissemination of cytokine throughout the tumour bed to engage with immune cells, simultaneously promoting prolonged retention in the tumour via the insertion of lipid tails of the conjugate in cell membranes in the local microenvironment (Fig. 5a). To test this, we prepared LbL NPs with covalently linked IL-12 using gel-phase fully saturated lipids for the liposome core (SAT LbL), which resist serum extraction, unlike the unsaturated lipids in our standard Mal LbL particles (Supplementary Table 2 and Extended Data Fig. 7a)^44–46^. SAT LbL NPs matched Mal LbL in size, charge, HM-1 binding and IL-12 bioactivity (Extended Data Fig. 7b–f), but showed substantially reduced lipid extraction and IL-12 release in serum (Extended Data Fig. 7g,h).

**Extended Data Fig. 8:**
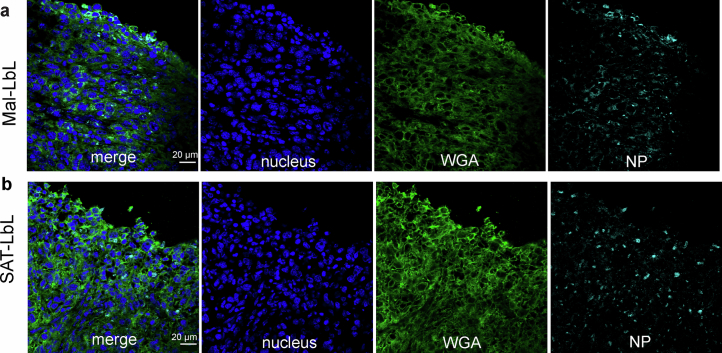
Confocal imaging of omentum tumor nodules shows both Mal-LbL and SAT-LbL penetrate tumor tissue. **a-b**, B6C3F1 mice were inoculated with 10^6^ HM-1-luc tumor cells on day 0 were administered fluorescently-tagged Mal-LbL of SAT-LbL NPs carrying 20 µg IL-12 on day 14 (*n* = 4 animals/group). One day after dosing, animals were sacrificed, and the omentum containing tumor nodules was frozen in optimal cutting temperature (OCT) compound then frozen sectioned and stained for confocal microscopy analysis. Shown are representative confocal images of omental tumor nodules from Mal-LbL (**a**) and SAT-LbL (**b**) treated animals.

To assess whether membrane composition affects lipid biodistribution, HM-1-tumour-bearing mice were treated with Mal LbL or SAT LbL NPs carrying fluorescent phosphatidylethanolamine (PE) lipids. One day later, omentum tumour nodules were examined by confocal microscopy. Both NP types penetrated the tumours efficiently (Extended Data Fig. 8a,b), but membrane composition altered the fluorescent lipid distribution: Mal LbL lipids localized to regions rich in cell membranes and extracellular matrix (wheat germ agglutinin (WGA) staining), whereas SAT LbL lipids clustered in WGA-poor pockets, suggesting the cellular internalization of SAT LbL particles and cargo (Fig. 5b,c).

We next carried out a therapeutic study administering a single dose of 20 µg of IL-12 as free cytokine, Mal LbL NPs, SAT LbL NPs or a control LbL NP lacking IL-12 (unloaded LbL; Fig. 5d). Unloaded NPs had no effect on tumour BLI or survival (Fig. 5e,f). Mal LbL NPs outperformed all other groups, dramatically reducing the tumour burden by day 3, delaying relapse until after day 22 and extending the median survival to 31 days. By contrast, free IL-12 and SAT LbL induced similar tumour regression with an earlier relapse by day 18 and shorter median survival of 27 days (Fig. 5e,f).

To validate these LbL NPs as a platform for cytokine delivery, we loaded an alternative cytokine to the NP–IL-15 superagonist (IL-15sa; Supplementary Fig. 5a) complex. IL-15sa was recently approved for bladder cancer^47^ and is effective in multiple preclinical models^48,49^. IL-15sa loading (~10% w/w) and bioactivity were comparable with IL-12 (Supplementary Fig. 5a–c). In metastatic HM-1 mice, Mal LbL (IL-15sa) NPs improved tumour control and survival versus free IL-15sa or UL NPs (Supplementary Fig. 5d–f). However, unlike IL-12-based treatments, Mal LbL (IL-15sa) only modestly extended the median survival (~20–25 days), indicating IL-12’s superior relevance for antitumour immunity in this model.

Having identified Mal LbL IL-12 NPs as the lead candidate, we evaluated its storage stability, which is critical for clinical translation. Mal LbL NPs retained a constant size and zeta potential after 1 month at 4 °C and 1 week at 22 °C (Supplementary Fig. 6a,b). Freezing in 5% dextrose preserved the particle properties after thawing (Supplementary Fig. 6c). IL-12 bioactivity remained unchanged across storage conditions in HEK-Blue reporter assays (Supplementary Fig. 6d). Thus, Mal LbL NPs demonstrate robust stability under clinically relevant storage conditions.

## Mal LbL NPs sensitize metastatic OC to ICI therapy

ICIs, such as antibodies blocking the negative regulatory receptors PD1 and CTLA4 expressed by T cells, are currently the most broadly effective class of immunotherapy agents clinically, but ICIs have failed to show substantial benefit in OC^50,51^. Responsiveness to checkpoint blockade correlates with the presence of pre-existing CD8 T cell infiltrates in patient tumours^52^, and thus, we hypothesized that enhanced T cell infiltration driven by LbL NPs could increase the responsiveness of OC to checkpoint inhibition. The strong IFN-γ expression induced by IL-12 in T cells and NK cells is expected to upregulate the expression of PD-L1 on tumour cells, a phenomenon termed adaptive resistance that has been shown to occur in mouse models of OC such as HM-1 and ID8 (refs. ^53,54^). Moreover, on activation, T cells express PD1 and prolonged IL-12 exposure induces TIM3 expression—both known inhibitory receptors^55–58^. We, thus, theorized that LbL NP treatment might sensitize OC to ICI therapy.

**Fig. 6:**
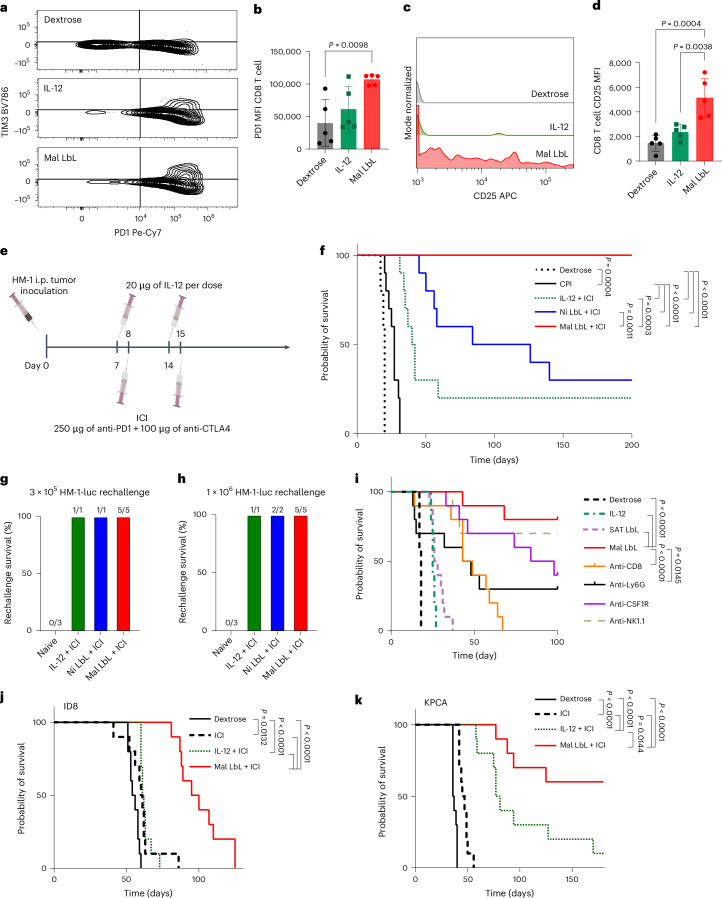
Two-dose Mal LbL NPs with checkpoint inhibitors eradicates metastatic OC. **a**–**d**, B6C3F1 mice (*n* = 5 animals per group) inoculated with 10^6^ HM-1 tumour cells on day 0 were treated on day 10 with 20 µg of IL-12 as a free cytokine or conjugated to Mal LbL NPs. Two days after dosing, ascites and i.p. tumour nodules (primarily omentum tissue) were harvested and processed for flow cytometry analysis. Flow plots of PD1 and TIM3 expression on T cells (CD45^+^CD3^+^) in tumour nodules (**a**), quantitation of PD1 MFI of T cells in tumour nodules (**b**), representative fluorescence histogram plots of CD25 expression on CD8 T cells (CD45^+^CD3^+^CD8^+^) in tumour nodules (**c**) and quantitation of CD25 MFI of CD8 T cells in tumour nodules (**d**). **e**,**f**, B6C3F1 mice (two cohorts with *n* = 5 animals per group per cohort) inoculated with 10^6^ HM-1-luc tumour cells on day 0 were treated on days 7 and 14 with 20 µg of IL-12 as a free cytokine or conjugated to Mal LbL or Ni LbL. Mice were also treated with 250 µg of anti-PD1 and 100 µg of anti-CTLA4 i.p. on days 8 and 15. Experimental timeline (**e**) and overall survival (**f**). On day 150, the surviving mice were rechallenged with either 3 × 10^5^ (**g**) or 10^6^ (**h**) i.p. HM-1-luc tumour cells. Percentage of mice per group that survived the rechallenge is shown. **i**, B6C3F1 mice (one cohort with *n* = 10 animals per group) inoculated with 10^6^ HM-1-luc tumour cells on day 0 were treated on days 7 and 14 with 20 µg of IL-12 as a free cytokine, Mal LbL, SAT LbL or Mal LbL in combination with depletion antibodies. With an exception of the dextrose group, mice were also treated with 250 µg of anti-PD1 and 100 µg of anti-CTLA4 via i.p. administration on days 8 and 15. Overall survival curves are shown. **j**,**k**, C57BL/6 mice (one cohort of *n* = 10 animals per group) were inoculated via i.p. administration with 5 × 10^6^ ID8 (*Trp53*^−*/*−^*; Brca2*^−*/*−^; **j**) or 10^6^ KPCA tumour cells (**k**) on day 0 were treated on days 7 and 14 with 20 µg of IL-12 as a free cytokine or conjugated to Mal LbL. Mice were also treated with 250 µg of anti-PD1 and 100 µg of anti-CTLA4 via i.p. administration on days 8 and 15. Statistics derived using all *n* from experiment with each animal as a data point. Overall survival curves are shown. *P* values were determined by one-way ANOVA followed by Tukey’s multiple comparison test (**b** and **d**) and the log-rank (Mantel–Cox) test (**f** and **i**–**k**). Source data

**Extended Data Fig. 9:**
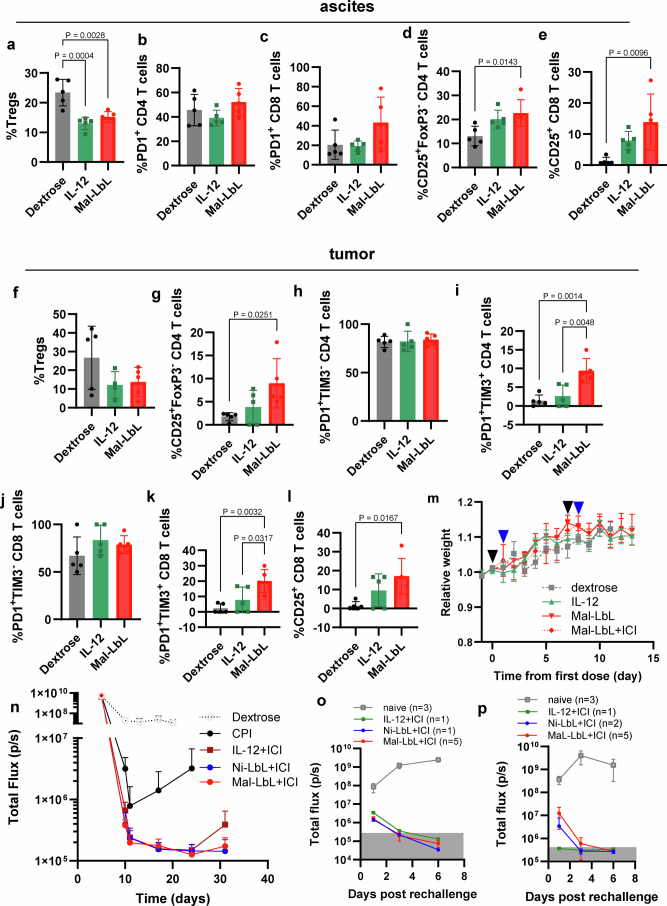
Mal-LbL NP treatment primes the TME for CPI treatment. **a-d** B6C3F1 mice (*n* = 5 animals/group) inoculated with 10^6^ HM-1 tumor cells on day 0 were treated on day 10 with 20 µg of IL-12 as a free cytokine or conjugated to Mal-LbL NPs. Two days after dosing ascites and i.p. tumor nodules (primarily omentum tissue) were harvested and processed for flow cytometry analysis. Shown are quantitation of the percentage of T regs among T cells in ascites (**a**), PD1 positive CD4 T cell (**b**) and CD8 T cell (**c**) in ascites, CD25^+^FoxP3^−^ of CD4 T cells (**d**) in ascites, CD25^+^of CD8 T cells (**e)** in ascites, T regs of T cells in tumor (**f**), CD25^+^FoxP3^−^ of CD4 T cells in tumor (**g**), PD1^+^TIM3^−^ of CD4 T cells in tumors (**h**), PD1^+^TIM3^+^ of CD4 T cells in tumors (**i**), PD1^+^TIM3^−^ of CD8 T cells in tumors (**j**), PD1^+^TIM3^+^ of CD8 T cells in tumors (**k**), and CD25^+^ of CD8 T cells (**l**). **m**, B6C3F1 mice (*n* = 10 animals/group) inoculated with 10^6^ HM-1-luc tumor cells on day 0 were treated on days 7 and 14 with 20 µg of IL-12 as a free cytokine or conjugated to Mal-LbL Mice were also treated with 250 µg of anti-PD1 and 100 µg of anti-CTLA4 i.p. on days 8 and 15. Shown are the relative body weight of mice (black arrows indicate IL-12 treatment and blue indicate CPI). None of the treated mice showed significant drop in bodyweight compared to dextrose control. **n-p**, B6C3F1 mice (2 cohorts with *n* = 5 animals/group/cohort) inoculated with 10^6^ HM-1-luc tumor cells on day 0 were treated on days 7 and 14 with 20 µg of IL-12 as a free cytokine or conjugated to Mal-LbL or Ni-LbL. Mice were also treated with 250 µg of anti-PD1 and 100 µg of anti-CTLA4 i.p. on days 8 and 15. Shown are the *in vivo* IVIS whole-animal i.p. BLI readings **(**mean ± s.d., **n)**. On day 150, surviving mice were rechallenged with either 3×10^5^ (**o**) or 10^6^ (**p**) HM-1-luc tumor cells i.p. Shown are the *in vivo* IVIS whole-animal i.p. BLI readings post rechallenge **(**mean ± s.d). In all panels, each point corresponds to a single animal, with values shown as mean ± standard deviation. Source Data

To better understand the T cell state and the effects of Mal LbL treatment, we performed an immunophenotyping protocol (Fig. 4e) focused on characterizing T cells for markers of activation (CD25, PD1 and TIM3) or the presence of Tregs (CD4^+^CD25^+^FoxP3^+^). In ascites, around 25% of T cells were Tregs, but both IL-12 or Mal LbL treatment reduced the proportion of Tregs (Extended Data Fig. 9a). Moreover, ~50% of CD4 T cells and 20%–25% of CD8 T cells expressed PD1 (Extended Data Fig. 9b,c). In particular, Mal LbL treatment increased the fraction of FoxP3^−^CD25^+^ CD4 effector T cells and CD25^+^ CD8 T cells compared with dextrose-treated mice (Extended Data Fig. 9d,e). In tumour nodules, both free IL-12 and Mal LbL NPs showed a trend towards reduced Tregs, which were ~10% of T cells in dextrose-treated mice (Extended Data Fig. 9f). Mal LbL treatment also increased CD25^+^FoxP3^−^ effector CD4 T cells fivefold (Extended Data Fig. 9g). Strikingly, in all groups, a majority (>75%) of both CD4^+^ and CD8^+^ tumour-infiltrating lymphocytes were PD1^+^TIM3^−^-activated effector cells, but only a small minority had a PD1^+^TIM3^+^-exhausted phenotype (Extended Data Fig. 9h–k). Treatment with Mal LbL NPs yielded a marked increase in PD1 expression compared with dextrose-treated mice (Fig. 6a,b). Further characterization of T cells in the tumour nodules revealed that the sustained IL-12 presentation by LbL NPs showed increased T cell activation based on elevated CD25 expression on CD8 T cells (Fig. 6c,d and Extended Data Fig. 9l).

Given the increased T cell levels and the expression of activation and inhibitory signals, we evaluated the treatment of HM-1-luc tumours with systemic anti-PD1 + anti-CTLA4 ICI therapy alone or combined with two weekly doses of IL-12 in free or NP form (Fig. 6e). Treatment with Mal LbL NPs and ICI via this scheme showed no sign of toxicity based on body-weight measurements in HM-1-luc mice (Extended Data Fig. 9m). The analysis of tumour burden via BLI clearly showed that ICIs alone could only mildly and transiently control the tumour growth (Extended Data Fig. 9n). On the other hand, there was a marked synergism when any form of IL-12 was included in the treatment, reducing tumour BLI to baseline for 1 month after tumour inoculation. ICI therapy alone had only marginal survival benefit over untreated controls, similar to what has been observed with OC in the clinic (Fig. 6f)^51^. Combining ICI with free IL-12 showed some efficacy, reducing the tumour BLI to baseline for ~25 days and leading to complete responses in 20% of the treated animals (Extended Data Fig. 9n and Fig. 6f). Ni LbL also synergized with ICI treatment, showing a significant extension in survival compared with free IL-12 and ICI, but ultimately resulted in only a 30% long-term survivor rate. Remarkably, however, Mal LbL showed a sensitization effect, achieving 100% cure with this treatment schedule (Fig. 6f). When challenged with fresh tumour cells on day 150, all Mal-LbL-NP-treated mice rejected the rechallenge (Fig. 6g,h and Extended Data Fig. 9o,p), demonstrating the induction of a strong memory response.

**Extended Data Fig. 10:**
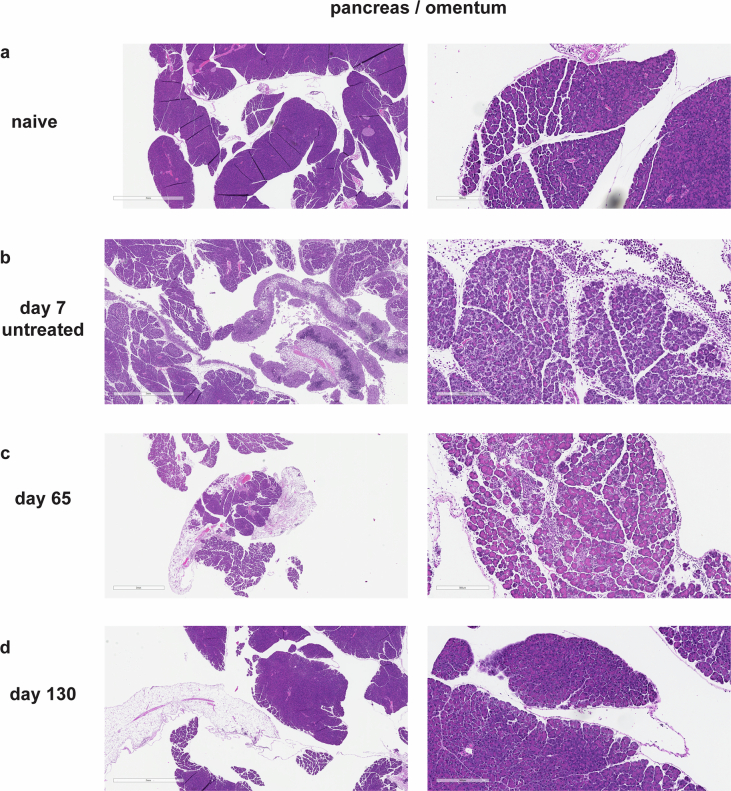
The combination of Mal-LbL NPs with CPI shows tumor clearance and recovery (day 130) of pancreatic findings. **a-d**, B6C3F1 mice inoculated with 10^6^ HM-1-luc tumor cells on day 0 were treated on days 7 and 14 with 20 µg of IL-12 as a free cytokine or conjugated to Mal-LbL Mice were also treated with 250 µg of anti-PD1 and 100 µg of anti-CTLA4 i.p. on days 8 and 15. Mice were euthanized before tumor inoculation (healthy *n* = 5), prior to the first dose (day 7, *n* = 3 animals), on day 65 (*n* = 5 animals), or on day 130 (*n* = 4 animals). Organs were harvested and processed for histopathologic evaluation. Shown are representative images of the primary finding in the omentum/pancreasin naive mice (**a**), day 7 mice showing tumor, inflammation and pancreatic degeneration (**b**), day 65 mice showing inflammation and pancreatic degeneration (**c**), and day 130 mice showing recovered omentum/pancreas (**d**).

To evaluate any long-term organ toxicity from the treatment, we euthanized HM-1-luc mice before (day 7) or after (days 65 and 130) Mal LbL and ICI combination treatment. H&E-stained sections of i.p. organs (liver, spleen, kidneys, intestines, omentum/pancreas, uterus and ovaries) were evaluated. On day 7, multiple nodules forming closely packed sheets of neoplastic cells were abutting most organs and were also present within the peritoneal adipose tissue (omentum and mesentery). Neoplastic cells were occasionally invading the liver capsule and encroaching into the liver parenchyma, causing some hepatocellular degeneration and necrosis. Pancreatic lobules were expanded by oedema, inflammation and neoplastic cells with mild acinar degeneration (Extended Data Fig. 10a,b). On day 65, clusters of mononuclear leucocytic infiltration, primarily lymphocytes and presumed degenerated neoplastic cells, were observed in adipose tissue (omentum/mesentery) and adjacent to all capsule and peritoneal serosal surfaces. The liver, spleen, intestines, kidneys, uterus and ovaries were unremarkable. The pancreas was the only organ in which mononuclear leucocytic infiltration was more prominent, dissecting the pancreatic nodules with occasional neutrophils and mild acinar degeneration (Extended Data Fig. 10c). On day 130, all tissues including the pancreas were histologically normal and no residual mononuclear cell infiltrates or apoptotic cells were observed, suggesting complete recovery in surviving mice (Extended Data Fig. 10d).

IL-12 exerts a multifaceted effect on immune cells of both innate and adaptive immunity either directly or through downstream cytokine signalling^59^. To understand the role of key immune cells and further validate the importance of lipid release from the targeted NPs for therapeutic efficacy, we repeated the combination treatment scheme of ICIs (Fig. 6e) with Mal LbL NPs in the presence of depletion antibodies against CD8 T cells (CD8α), NK cells (NK1.1), neutrophils (Ly6G) and macrophages (CSFR1) in the HM-1 model, with free IL-12 and SAT LbL NPs as additional control groups. Similar to IL-12 monotherapy, SAT LbL NPs in combination with ICIs did not outperform free IL-12 with ICIs (Fig. 6i). Antibody-mediated depletion revealed an early dependence on neutrophils and strong dependence on CD8 T cells for tumour control (Fig. 6i). Depletion of NK cells or macrophages did not show major effects in treatment, although macrophage depletion appeared to reduce long-term survivor rates.

On the basis of the strong efficacy of Mal LbL/ICI combination therapy in treating HM-1 tumours, we evaluated this treatment in additional models of OC peritoneal metastasis: ID8 tumours^60^ modified to carry *Trp53*^−*/*−^ and *Brca2*^−*/*−^ mutations^61^ modelling OC with DNA homologous repair deficiency; and KPCA tumours^62^ carrying driver mutations Kras^G12N^, p53^R172H^, Ccne1^OE^ and Akt2^OE^, modelling DNA homologous repair proficient tumours. Both these models are resistant to checkpoint blockade^62–64^. In the ID8 model, ICI alone or ICI combined with free IL-12 had minimal efficacy, with a median survival of 55–60 days (Fig. 6j). On the other hand, the combination of Mal LbL with ICI led to a major increase in median survival to 98 days. No complete responses were observed, potentially due to the low immunogenicity of the ID8 model, which is thought to lack the expression of neoantigens that bind to the mouse major histocompatibility complex^65,66^. Similar to the ID8 model, treatment of the KPCA model with ICI elicited very minor benefits on overall survival (Fig. 6k). Unlike the ID8 model, free IL-12 combined with ICI increased the median survival to 79 days with 10% long-term survivors. However, when we delivered IL-12 using Mal LbL in combination with ICI, 60% of the treated mice exhibited complete responses. This is a notable result as the KPCA model has been well characterized to have an immunosuppressive TME and low T cell infiltration^62^. Taken together, these results demonstrate that engineering the rapid targeting and slow local release of IL-12 in ovarian tumour nodules using LbL NPs substantially sensitizes tumours to clinically approved ICI combination therapy.

## Outlook

Cytokine therapies have faced notable challenges in development and regulatory approval^9,67^. IL-12, despite its strong potential to drive antitumour immunity, has failed clinically due to a low therapeutic index when administered as a free protein^59^. Here we report the use of LbL NPs that target and stably attach to cancer cell membranes, enabling localized and prolonged presentation of IL-12 in peritoneally disseminated metastatic OC (a comparison with alternative approaches is provided in Supplementary Note). We demonstrate that optimized LbL IL-12 NPs are non-toxic, elicit strong systemic antitumour immunity, drive remodelling of the TME and strongly sensitize ovarian tumours to ICI therapy. In particular, this remodelling includes a marked increase in CD8^+^ T cell infiltration and local activation, changes that directly correlate with improved systemic tumour-specific immunity. Similar to previous IL-12 therapies^9,48,68^, treatment relied heavily on CD8 T cells but also showed an early neutrophil response, aligning with their role in tumour control during TME remodelling^69^. Importantly, unlike our prior LbL NPs for cytokine delivery based on the Ni LbL chemistry (which reduced toxicity but only showed marginal enhancement of therapeutic efficacy^18,19^), Mal LbL NPs were found to increase efficacy and dramatically sensitize ovarian tumours to ICI. Importantly, we demonstrate that the efficacy of LbL IL-12 NPs is dependent on both covalent conjugation of the cytokine payload to lipids and lipid extraction, a known but relatively unexplored property of lipid-based NPs. It is also clear that targeting the LbL NPs to the tumour cell membrane surface is a critical aspect of this approach as UL NPs yielded similar results to free cytokine. In addition to delayed lipid conjugate release from the NP, such a mechanism may promote the trafficking of therapeutic payloads to tumour-draining lymph nodes^70^.

In summary, this study demonstrates that engineering PLE-coated LbL NPs carrying IL-12 can facilitate both tumour-targeted delivery and sustained cytokine release within disseminated tumour nodules. Combining tumour targeting with localized cytokine dissemination in the TME significantly improves the efficacy of this immunotherapy against metastatic OC. Importantly, optimized LbL NPs exhibited strong synergy with checkpoint blockade, the current gold standard for cancer immunotherapy in the clinic.

## Methods

### Materials

1,2-Sistearoyl-*sn*-glycero-3-phosphocholine (DSPC), 1,2-dioleoyl-*sn*-glycero-3-[(*N*-(5-amino-1-carboxypentyl)iminodiacetic acid)succinyl] (nickel salt) (18:1 (Ni)NTA-DGS), 1,2-dioleoyl-*sn*-glycero-3-phosphoethanolamine-*N*-[4-(*p*-maleimidophenyl)butyramide] (sodium salt) (18:1 MPB-PE), 1-palmitoyl-2-oleoyl-*sn*-glycero-3-phospho-(1′-*rac*-glycerol) (sodium salt), 1,2-dioleoyl-*sn*-glycero-3-phosphoethanolamine-*N*-dibenzocyclooctyl, 1,2-distearoyl-*sn*-glycero-3-phospho-(1′-*rac*-glycerol) (sodium salt), 1,2-dipalmitoyl-*sn*-glycero-3-phosphoethanolamine-*N*-[4-(*p*-maleimidophenyl)butyramide] (sodium salt) (16:0 MPB-PE), 1,2-dipalmitoyl-*sn*-glycero-3-phosphoethanolamine-*N*-dibenzocyclooctyl (16:0) and cholesterol were purchased from Avanti Polar Lipids. PLR with a molecular weight of 9.6 kDa and PLE with a molecular weight of 15 kDa were purchased from Alamanda Polymers. BDP TMR azide (Lumiprobe) and BDP 630/650 azide (Lumiprobe) were conjugated to 1,2-dioleoyl-*sn*-glycero-3-phosphoethanolamine-*N*-dibenzocyclooctyl or 1,2-dipalmitoyl-*sn*-glycero-3-phosphoethanolamine-*N*-dibenzocyclooctyl (18:0) in chloroform to generate fluorescently labelled lipids. Successful conjugation was validated via thin-layer chromatography, which indicated <1% free dye. For immunophenotyping (all mouse targets), antibodies for CD3e (clone 145-2C11, FITC and BUV805), CD49b (clone DX5, PE), CD45 (clone 30-F11, PE-Cy5.5 and BUV496), F4/80 (clone T45-2342, PE), CD4 (clone GK1.5, BUV 563), CD80 (clone 16-10A1, FITC), CD11b (clone M1/70, FITC) and Ly6C (clone AL-21, PE) were purchased from BD Biosciences. Antibodies for CD4 (clone GK1.5, APC-Cy7), CD8α (clone 53-6.7, PE), CD206 (clone C068C2, PE-Cy5), CD279 (PD1, clone 29 F.1A12, PE-Cy7), CD366 (Tim3, clone RMT3-23, BV785), CD25 (clone PC61, BV-421) and Ly6G (clone 1A8, APC) were purchased from BioLegend. Antibodies for CD8α (clone KT15, FITC) and FoxP3 (clone FJK-16s, PE) were purchased from Invitrogen. Ultrapure diH_2_O was obtained through a Milli-Q water system (EMD Millipore).

### Animals

All animal work was conducted under the approval of the Massachusetts Institute of Technology Division of Comparative Medicine Institutional Animal Care and Use Committee in accordance with federal, state and local guidelines (protocols numbers 2303000488 and 082105224). B6C3F1 mice were purchased from Jackson and Charles River Laboratories, whereas C57BL/6 mice were purchased from Charles River Laboratories. Female mice were used between 8–12 weeks of age, unless otherwise noted. Mice were evaluated for signs of morbidity or weight loss and euthanized if body weight decreased by ≥20% or if the body condition score dropped below 2. No statistical methods were used to predetermine the sample sizes, but our sample sizes are similar to those reported in previous publications^9,19^. For therapeutic studies in tumour-bearing mice, animals were randomized into groups with comparable tumour burdens.

### Cell culture

OV2944-HM-1 cells were acquired through Riken BRC and were cultured in α-MEM. HEK-Blue IL-12 and HEK-Blue IL-2 (InvivoGen) cells were cultured and used for IL-12 and IL-15sa bioactivity assessment, respectively, according to the manufacturer’s instructions. ID8 (Trp53^−^/Brca2^−^) were obtained from Iain McNeish’s laboratory at the University of Glasgow and cultured in Dulbecco’s modified Eagle’s medium^61^. Cell media were also supplemented with 10% fetal bovine serum (FBS) and 1% penicillin–streptomycin, with cells incubated in a 5% carbon-dioxide-humidified atmosphere at 37 °C. KPCA cells were obtained from Weinberg’s laboratory and cultured with 4% heat-inactivated FBS, 1% of insulin–transferrin–selenium (Thermo Fisher Scientific) and 1% of penicillin–streptomycin^62^. All cell lines were murine pathogen tested and confirmed to be mycoplasma negative by Lonza MycoAlert Mycoplasma Detection Kit.

### Recombinant single-chain IL-12 production

Single-chain IL-12 sequence^71^ was synthesized as a genomic block (Integrated DNA Technologies) and cloned into the gWIZ expression vector (Genlantis). For conjugation with maleimides, a C-terminus cysteine was added after the polyhistidine tag. Plasmids were transiently transfected into Expi293 cells (Thermo Fisher Scientific). After 5 days, cell culture supernatants were collected and protein was purified in an ÄKTA pure chromatography system using HiTrap HP Niquel sepharose affinity column, followed by size exclusion using Superdex 200 Increase 10/300 GL column (GE Healthcare Life Sciences). For the production of IL-15sa, the murine version of dimeric IL-15N72D:IL-15RαSu/Fc complex was designed based on ref. ^72^. A terminal 6×His-tag was added for purification purposes with a C-terminal cysteine for conjugation. The open reading frame was synthesized as a genomic block (Integrated DNA Technologies) and cloned into the gWIZ expression vector (Genlantis). The same transfection and purification protocol was used as IL-12. Endotoxin levels in purified protein were measured using Endosafe Nexgen-PTS system (Charles River) and assured to be <5 EU mg^−1^ of protein.

### Liposome synthesis

A lipid solution was prepared by mixing (all mol%) 65% of DSPC (25 mg ml^−1^), 24% of cholesterol (25 mg ml^−1^), 6% of 1-palmitoyl-2-oleoyl-*sn*-glycero-3-phospho-(1′-*rac*-glycerol) (25 mg ml^−1^) and 5% of either 18:1 (Ni)NTA-DGS (5 mg ml^−1^) or 18:1 MPB-PE (5 mg l^−1^) and then formed into a thin film using a rotary evaporator (Buchi). Lipid films were allowed to further dry overnight in a desiccator, and then hydrated at 0.5–1 mg ml^−1^ using diH_2_O and sonicated for 3–5 min at 65 °C and then extruded (Avestin Liposofast LF-50) once at 65 °C through a 100-nm membrane (Cytiva Nuclepore) followed by extrusion three times through 50-nm membranes (Cytiva Nuclepore). The extruded liposomes were added to an ice bath. SAT NPs were generated with the same procedure as Mal NPs, but its composition (all mol%) was 65% of DSPC (25 mg ml^−1^), 24% of cholesterol (25 mg ml^−1^), 6% of 1,2-distearoyl-*sn*-glycero-3-phospho-(1′-*rac*-glycerol) (25 mg ml^−1^) and 5% of 16:0 MPB-PE (5 mg ml^−1^). Lipids were stored at –20 °C in amber vials in chloroform, except 1,2-distearoyl-*sn*-glycero-3-phospho-(1′-*rac*-glycerol) that was stored in a 1:1 (v/v) mixture of chloroform and methanol instead of pure chloroform.

For coupling of scIL-12 via Ni:His-tag interactions, scIL-12 was added to 0.5 mg ml^−1^ of (Ni)NTA-DGS liposomes at a molar ratio of 28:1 of (Ni)NTA-DGS lipids to IL-12. After incubation with IL-12 at 4 °C for 18 h, Ni UL liposomes were purified via tangential flow filtration on a 100-kDa modified polyethersulfone membranes (Repligen) against six diafiltration volumes of diH_2_O.

For the covalent linkage of scIL-12 to Mal liposomes, the solution pH of MPB-PE liposomes was adjusted to pH 5 with hydrochloric acid before lipid-film hydration. Following membrane extrusion, liposomes at 0.33 mg ml^−1^ were adjusted to pH 7.0 with 10-mM HEPES followed by the addition of scIL-12 containing a terminal cysteine residue at a molar ratio of 25:1 of MPB-PE lipid to protein for at least 12 h at 4 °C in a rotating mixer. Any remaining maleimides were quenched with a 100-fold molar excess of L-cysteine (Sigma) for 1.5 h on ice. IL-15sa coupling was performed with the same procedure, but particles were diluted to 0.16 mg ml^−1^ during coupling, and the molar ratio of MPB-PE to protein was 20:1.

For the fluorescent labelling of liposomes, 0.2 mol% of DSPC was replaced by either DOPE-TMR or DOPE-630/650. IL-12 concentrations were measured via ELISA (Peprotech) and the lipid content was quantified via the Stewart assay^73^. For IL-15sa quantification, we captured the construct with anti-IL-15 Ab (Peprotech) and then detected the protein based on its Fc region using anti-IgG Ab (Thermo Fisher).

### LbL film deposition onto NPs

The assembly of polyelectrolyte layers was performed by adding UL particles to a diH_2_O solution with 0.3–0.4 weight equivalents of PLR relative to the lipid in a glass vial under sonication and incubating on ice for at least 30 min. Excess PLR polymer was purified by tangential flow filtration through a 100-kDa modified polyethersulfone membrane (Repligen) pretreated with a 10 mg ml^−1^ solution of free PLR. For the terminal PLE layer, purified particles coated with PLR were added to a diH_2_O solution with PLE in a glass vial under sonication at one weight equivalent of polymer to lipid. LbL particles were then purified by tangential flow filtration on a separate 100-kDa modified polyethersulfone membrane (Repligen) to remove any excess PLE.

### Characterization of particle preparations

Dynamic light scattering and zeta potential measurements were made on a Zetasizer Nano ZSP (Malvern). NP micrographs were acquired via transmission electron microscopy on a JEOL 2100F microscope (200 kV) with a magnification range of ×10,000–60,000. All the images were recorded on a Gatan 2k × 2k UltraScan charge-coupled device camera. Negative-stain sample preparation was performed by adding 10 µl of NPs on a 200-mesh copper grid coated with a continuous carbon film and allowing for sample adsorption for 60 s. Excess solution was then removed by touching the grid with a Kimwipe. The grid was then quickly washed by adding 10 µl of negative-staining solution, phosphotungstic acid, 1% aqueous solution, and then removing excess by touching the grid with a Kimwipe. Then, the grid was mounted on a JEOL single-tilt holder equipped in the transmission electron microscopy column for image capture. For IL-12 detection staining, NP samples were deposited on grids for 5 min and then washed twice with diH_2_O. Grids were blocked with 1% bovine serum albumin (BSA) for 5 min, washed thrice with phosphate-buffered saline (PBS) and then incubated with 10 µg ml^−1^ of anti-IL-12 Ab (BioLegend, clone C15.6) in 0.5% BSA for 1 h. Grids were then washed four times with 0.5% BSA and then incubated with 40× diluted Nanogold-IgG goat anti-rat IgG (Nanoprobes) for 30 min. Grids were then washed twice with 0.5% BSA, thrice with PBS, thrice with diH_2_O, stained with 0.2% uranyl acetate for 5 min and then washed thrice with diH_2_O.

### Fluorescent labelling of polymers

PLR was solubilized at 100 mg ml^−1^ in diH_2_O and then mixed with two molar equivalents of BDP-TR-NHS-ester (Lumiprobe) in dimethyl sulfoxide (DMSO) to generate a 15 mg ml^−1^ PLR solution. The reaction was catalysed with ten molar equivalents of triethylamine and allowed to react for 4 h at room temperature and then overnight at 4 °C. PLR-TR was purified via reverse-phase high-pressure liquid chromatography on a Jupiter C4 column (5-µm particles, 300 Å; Phenomenex) using a water:acetonitrile gradient that started at 20% actetonitrile for 5 min and then increased to 35% in a linear gradient until 10 min. Isocratic elution at 35% was performed for 30 min and then the elution buffer was increased to 95% to clean out the column for 10 min and then dropped back to 20% acetonitrile to re-equilibrate the column for 5 min. The collected purified PLR-TR fractions were then diluted tenfold with diH_2_O and then lyophilized. PLE at 10 mg ml^−1^ was labelled by reacting with five molar equivalents of sulfo-cyanine3 NHS ester (Lumiprobe) in PBS adjusted to pH ~8.5 with 0.1 M of sodium bicarbonate. Excess dye was removed via extensive 0.9 wt% NaCl dialysis followed by extensive diH_2_O dialysis using a 3-kDa regenerated cellulose membrane (Repligen) and the purified PLE-cy3 was lyophilized until use.

### Analysis of LbL film stability

For PLR stability, PLR/PLE films were assembled onto Mal UL NPs as described in the ‘LbL film deposition onto NPs’ section, but the PLR solution was doped with 30% PLR-TR. For PLE stability, the PLE solution was doped with 50% PLE-cy3. Particles were incubated in various buffer solutions at 0.1 mg ml^−1^ in a shaker at 37 °C. At defined intervals, aliquots were extracted from the incubation solution and free polymers were separated from the NP. For PLE separation, samples were spun on a 300-kDa centrifugal filter (VivaSpin500, Sartorius) at 10*g* for 15 min and the permeate fluorescence was compared with the fluorescence of the initial sample. For PLR separation, the NPs in the sample were pelleted by centrifuging at 25,000*g* for 30 min and the supernatant PLR fluorescence was compared with the initial sample PLR fluorescence. Particles were validated to lack free polymers by centrifuging in diH_2_O. Fluorescence was measured on 96-well plates using a plate reader (Tecan Infinite 200). Ascites for release experiments were collected 2 weeks after i.p. injection of 10^6^ OV2944-HM-1 cells in PBS into B6C3F1 mice.

### IL-12 accessibility via monoclonal antibody binding

IL-12 accessibility to monoclonal antibody binding was determined by using the antibodies in an IL-12 sandwich ELISA kit (Peprotech). After coating a 96-well plate with anti-IL-12 antibodies and blocking the plate with BSA according to the manufacturer’s protocol, Mal UL or Mal LbL NPs were captured onto the plate by incubating the samples for 2 h in either diH_2_O or HEPES-buffered saline solution supplemented with 10% FBS. After sample incubation with the capture antibodies, the plates were washed and the total captured IL-12 was determined following the manufacturer’s protocol.

### In vitro cellular association

HM-1 cells were plated on a tissue-culture 96-well plate at a density of 50,000 cells per well. The next day, wells were dosed with NPs and left for the target incubation time (4 h or 24 h). For analysis via flow cytometry, NPs were dosed at 0.02 mg ml^−1^ and allowed to incubate with cells for 4 h at 37 °C. Cells were washed with PBS and then detached from the plates using 0.25% trypsin and stained with DAPI (15 min of incubation) for viability assessment and fixed with 2% paraformaldehyde (30 min of incubation) until analysis by flow cytometry using an LSRFortessa (BD Biosciences). For the assessment of NP-associated fluorescence in a fluorescence plate reader, a dose of 0.05 mg ml^−1^ was used. Before cell washing with PBS, the supernatant was removed from the well and diluted ten times with DMSO. Cells were then washed three times with PBS and disrupted with DMSO. NP fluorescence associated with cells was then normalized to the supernatant fluorescence. The relative fluorescence of each formulation was then compared with a UL liposome control containing the same fluorophore. For confocal imaging, eight-well chambered coverglasses (Nunc Lab-Tek II, Thermo Scientific) were coated with rat tail collagen type I (Sigma-Aldrich) per the manufacturer’s instructions. HM-1 cells were plated into the wells at a density of 10,000 per well and left to adhere overnight before NP treatment. After the desired incubation time with NPs, cells were washed thrice with PBS. After washing, cells were fixed in 4% paraformaldehyde for 10 min and then washed (thrice with PBS) and stained with WGA conjugated to Alexa Fluor 488 (Invitrogen) and Hoechst 33342 (Thermo Scientific) following the manufacturers’ instructions. Images were analysed using FIJI ImageJ (version 1.54f)^74^. Slides were imaged on an Olympus FV1200 laser scanning confocal microscope.

### Fluorescent labelling of IL-12

IL-12 was labelled with indocyanine green tetrafluorophenyl ester (AAT Bioquest) by solubilizing the dye in dimethyl sulfoxide at 1 mg ml^−1^ and adding it to IL-12 at 3 mg ml^−1^ in PBS supplemented with 0.1 M of sodium bicarbonate at a dye-to-protein molar ratio of 1.2:1. Excess dye was removed via 7-kDa desalting columns (Zeba Spin, Thermo Fisher) and validated via thin-layer chromatography.

### IL-12 release assay

IL-12 release from liposomes was quantified using the same procedure as the quantification of PLE stability.

### Serum-induced lipid exchange

Lipid release from NPs was assessed by generating liposomes with a high density (1 mol%) of DOPE-630-650 (for Mal LbL) or DPPE-630/650 (for SAT LbL) to induce fluorescence quenching. Particles were then mixed with 100% FBS solution supplemented with penicillin–streptomycin and incubated in 96-well plates in a shaker at 37 °C. At certain intervals, dye fluorescence was measured (excitation, 610 nm; emission, 650 nm) on a plate reader (Tecan Infinite 200) and compared with the total dye fluorescence, which was obtained by dissolving the NPs in DMSO.

### Kinetics of NP association with high-tumour-burden tissues in metastatic OC model

B6C3F1 mice were inoculated with HM-1-luc cells through the i.p. injection of 10^6^ cells in PBS. For kinetics of LbL NP association, 2 weeks after tumour inoculation, mice were injected with NPs containing fluorescently labelled lipid (DOPE-630/650, 0.2 mol%) and euthanized 1, 2, 4, 12 and 24 h after dosing. UGT and omentum tissues were extracted and placed in PBS under ice. Tissues were then transferred to IVIS (PerkinElmer) to quantify ex vivo tissue NP fluorescence. Data were analysed using Living Image software (version 4.7.2). Background fluorescence measurements were made for each organ based on the signal from mice treated with dextrose and measurements were normalized to the tissue weight.

### Pharmacokinetic and biodistribution in metastatic OC model

B6C3F1 mice were inoculated with HM-1-luc cells through the i.p. injection of 10^6^ cells in PBS. Two weeks after tumour inoculation, mice were injected with NPs containing fluorescently labelled lipid (DOPE-630/650, 0.2 mol%) and IL-12 indocyanine green. The same IL-12 indocyanine green was used for all groups to avoid labelling efficiency differences, and groups were dosed intraperitoneally with 20 µg of IL-12. In vivo tumour radiance efficiency was measured on IVIS by imaging the mice i.p. region. After the final time point (4 h or 24 h), mice were euthanized, and the ascites and organs were removed and placed in PBS under ice. Organs were then transferred to a 1 mg ml^−1^ solution of D-luciferin in PBS and incubated for 5 min and then placed in IVIS to determine each organ’s BLI, NP fluorescence and IL-12 fluorescence. Data were analysed using Living Image software. Background fluorescence measurements were made for each organ based on the signal from mice treated with dextrose. For correlation analysis, the weight-normalized bioluminescence flux (photons s^−1^ g^−1^) and radiance efficiency ((photons s^−1^)/(µW cm^−2^) g^−1^) for each organ were analysed on GraphPad Prism 9 for their correlation via the Pearson’s correlation coefficient. The concentrations of IL-12 and IFN-γ in ascites fluid were determined via ELISA (Peprotech).

### IVIS image pixel correlation analysis

IVIS images were extracted using the Living Image software in the same range of BLI and IL-12 fluorescence values. Pixel intensity values for all tissues of each mouse were extracted using ImageJ and then analysed for Spearman’s correlation between BLI and IL-12 using GraphPad Prism 9.

### Comparison of NP association in healthy and tumour-burdened tissues

B6C3F1 mice were inoculated intraperitoneally with 10^6^ cells of HM-1 in PBS. Two weeks after tumour inoculation, healthy B6C3F1 or the tumour-bearing mice were treated with 20 µg of IL-12 in fluorescently labelled Mal LbL or Mal UL NPs. Four hours after dosing, the organs were harvested, their NP fluorescence was quantified on IVIS and then weighted. Background fluorescence measurements were made for each organ based on the signal from mice treated with dextrose. To account for variation in peritoneal retention between healthy and tumour-bearing mice, the fraction of recovered NP fluorescence was calculated as described previously^17,18^.

### Cryogenic freezing of omentum tumours

Omentum tissue from the biodistribution study was embedded in optimal cutting temperature (OCT) compound and rapidly frozen in cryo-moulds using isopentane with dry ice. Samples were sectioned at 10 µm with a microtome cryostat and placed onto Tissue Path Superfrost Plus Gold Slides (Fisherbrand) and stored at –80 °C. For staining, slides were rapidly fixed with ice-cold 4% methanol-free formaldehyde for 10 min and then washed with PBS and blocked with 10% goat serum for 1 h. The samples were then incubated with PE anti-IL-12/IL-23 p40 antibody (BioLegend) overnight at 4 °C in 1% BSA PBS buffer. After overnight incubation, Hoechst 33342 (Thermo Fisher) and WGA-Alexa Fluor 488 (Thermo Fisher) were added and allowed to incubate for 30 min at room temperature. Samples were then washed with PBS and then mounted with a coverslip using ProLong Gold (Thermo Fisher) and stored at 4 °C after drying. Slides were imaged on an Olympus FV1200 laser scanning confocal microscope.

### Immunophenotyping via flow cytometry and blood panel analysis

B6C3F1 mice were inoculated intraperitoneally with 10^6^ cells of HM-1 in PBS. Ten days after tumour inoculation, mice were treated with either dextrose (vehicle control) or 20 µg of IL-12 in free, Mal UL or Mal LbL formats. Two days after dosing, mice were bled retro-orbitally and then euthanized to extract ascites cells via peritoneal lavage with PBS. Peritoneal tumour nodules and spleen were also collected. Part of the blood samples were submitted to The Division of Comparative Medicine at MIT to perform a complete blood count and analysis of liver function and the remainder was processed with ACK lysing buffer (Gibco) to isolate peripheral blood mononuclear cells (PBMCs). Spleens were processed on a 70-µm cell strainer with a syringe plunger and then exposed to the ACK lysing buffer to lyse red blood cells. Tumour nodules from each mouse were diced with scissors and then incubated for 1 h at 37 °C in 2 ml of 1 mg ml^−1^ collagenase type IV (Sigma) in RPMI media. After collagenase incubation, tumours were processed on a 70-µm cell strainer and then collected with an insulin syringe into Falcon tubes to pellet tumour cells and wash out the collagenase solution. For cell staining with antibodies, samples were placed in 96-well plates, then centrifuged and resuspended in Fc block solution (BD Biosciences) for 5 min. Freshly prepared antibody panels were then mixed with the samples and allowed to react for 20 min. Finally, DAPI (2 µg ml^−1^, BD Biosciences) was added to each well and allowed to react for 5 min. Stained cells were washed twice with flow cytometry buffer (PBS with 0.5% BSA and 2 mM of EDTA), then resuspended in 2% paraformaldehyde in PBS for 30 min, washed and stored at 4 °C for analysis the next day on a flow cytometry instrument (LSRFortessa, BD Biosciences). Flow cytometry buffer was used to prepare the Fc block and antibody solutions. For intracellular staining, the Foxp3/Transcription factor staining kit (eBiosciences) was used following the manufacturer’s instructions with Zombie NIR (BioLegend) used instead of DAPI for live/dead staining according to the manufacturer’s instructions. The gating strategy for flow cytometry analysis with each antibody panel used are shown in Supplementary Figs. 7 and 8.

### Efficacy studies with metastatic OC model

B6C3F1 mice were inoculated intraperitoneally with 10^6^ cells of HM-1-luc in PBS. C57BL/6 were inoculated with either 10^6^ cells of KPCA.B^62^ cells of 5 × 10^6^ ID8 (*Trp53*^−*/*−^*, Brca2*^−*/*−^)^61^ cells in PBS. One week after inoculation, treatment with either IL-12 or IL-15sa was initiated, as indicated in each figure. All cytokine treatments were of either 20 µg of IL-12 or 10 µg of IL-15sa. For chemotherapy experiments, mice were treated via i.p. administration with 40 mg kg^−1^ carboplatin (in 5% glucose) and 10 mg kg^−1^ paclitaxel (in 0.9% sodium chloride). For combination with ICIs, mice received 250 µg of anti-PD1 antibody (clone 29 F.1A12, BioXCell) and 100 µg of anti-CLTA4 antibody (clone 9D9, BioXCell) via i.p. administration 1 day after treatment with IL-12 constructs. Animal weights were tracked daily after treatments for signs of toxicity. Bioluminescence was measured on a IVIS 10 min after i.p. injection of 3 µg of D-luciferin sodium salt (GoldBio) before the start of the treatments to normalize the groups and up to 30 days after tumour inoculation or as needed to quantify the tumour burden.

### Tracking of serum cytokine levels

B6C3F1 mice were inoculated intraperitoneally with 10^6^ cells of HM-1-luc in PBS and then treated with either dextrose or 20 µg of IL-12 as free form or conjugated to Mal LbL on days 7 and 14. Blood was collected retro-orbitally the day before as well as 24, 48 and 72 h after each treatment. Serum was isolated on clotting activator/gel tubes (Sarstedt) according to the manufacturer’s instructions and stored at –20 °C until quantification of IL-12 and IFN-γ via ELISA (Peprotech).

### Antibody-mediated cellular depletion

Immune cell depletions were carried out with antibodies targeting CD8a (BioXCell, clone 2.43, 400 μg twice weekly), NK1.1 (BioXCell, clone PK136, 400 μg twice weekly) and CSF1R (BioXCell, clone AFS98, 300 μg every other day), as previously described^9,48^. All depletions were given via i.p. administration in 100 μl of PBS. Depletions were initiated 1 day before treatment and carried out for 4 weeks. Depletions were carried out in HM-1-luc-bearing mice treated with 20 µg of Mal LbL on days 7 and 14 and 250 µg of anti-PD1 antibody and 100 µg of anti-CLTA4 antibody on days 8 and 15.

### IFN-γ ELISpot

Blood was collected from mice via submandibular bleeding and lysed in ACK Lysis Buffer and then placed in RPMI supplemented with 10% FBS, 1% penicillin–streptomycin, 1× non-essential amino acids (Invitrogen), 1× sodium pyruvate (Invitrogen) and 1× 2-mercaptoethanol (Invitrogen). On the same day, HM-1-luc cells (treated with 500 U ml^−1^ of IFN-γ overnight) were subjected to 120 Gy of radiation and trypsinized into a single-cell suspension in the same supplemented RPMI. Then, 25,000 irradiated HM-1-luc cells were mixed with 3 × 10^5^ PBMCs per sample and seeded in a 96-well ELISpot plate (BD Biosciences) that was precoated with IFN-γ capture antibody (BD Biosciences). Plates were cultured for 24 h in a 37 °C incubator and then developed according to the manufacturer’s protocol. Plates were scanned using a CTL ImmunoSpot Plate Reader, and data were analysed using CTL ImmunoSpot Software (version 5.1.36).

### Toxicity assessment

For acute toxicity assessment, B6C3F1 mice were inoculated intraperitoneally with 10^6^ cells of HM-1-luc in PBS, and then treated on day 10 post-inoculation with either dextrose (vehicle control) or 20 µg of IL-12 in free, Mal UL or Mal LbL formats. Mice were euthanized on day 2 post-dose. For long-term treatment effects, B6C3F1 mice were inoculated intraperitoneally with 10^6^ cells of HM-1-luc in PBS and treated with 20 µg of Mal LbL on days 7 and 14 and 250 µg of anti-PD1 antibody and 100 µg of anti-CLTA4 antibody on days 8 and 15. Mice were euthanized before (on day 7) or after (days 65 and 130 post-inoculation) treatment. Organs (liver, spleen, omentum/pancreas, kidneys, intestines, uterus and ovaries) were harvested into cold RPMI media, sectioned and transferred into tissue cassettes and then fixed in 4% paraformaldehyde for at least 24 h and then transferred to 70% ethanol and immediately processed and paraffin embedded to generate H&E-stained tissue/slides. Histopathology assessment was performed in consultation with a board-certified veterinary pathologist.

### Storage stability of particles

Mal LbL NPs loaded with IL-12 were prepared as described. Aliquots at 1 mg ml^−1^ in diH_2_O water were stored at either 4 °C for 1 month or 22 °C for 1 week. Samples were measured at least every other day for size and zeta potential in the first week and then at least weekly for 1 month. For frozen storage, 1 mg ml^−1^ samples were made with 5% dextrose and frozen at –20 °C. Frozen samples were characterized for retention of their size and zeta potential 1 day or 1 week after freezing. IL-12 bioactivity was quantified via the HEK-Blue IL-12 reporter cell lines at select time points and compared with fresh Mal LbL NPs.

### Statistical analysis

GraphPad PRISM 9 was used to perform the statistical analyses. Comparisons between two groups was performed via unpaired *t*-tests. For multiple groups or multiple variables analysis, one-way, or two-way ANOVAs were used with Tukey’s post hoc correction for time-based analysis or Šidák post hoc for other ANOVA analysis. Data distribution was assumed to be normal, but this was not formally tested. Data collection and analysis were not performed blind to the conditions of the experiments. No data points were subjectively excluded from the statistical analysis.

### Reporting summary

Further information on research design is available in the Nature Portfolio Reporting Summary linked to this article.

## Online content

Any methods, additional references, Nature Portfolio reporting summaries, source data, extended data, supplementary information, acknowledgements, peer review information; details of author contributions and competing interests; and statements of data and code availability are available at 10.1038/s41563-025-02390-9.

## Supplementary information

Supplementary Information Supplementary Figs. 1–8, Tables 1 and 2 and Note.

Reporting Summary

## Source data

Source Data Figs. 1–6 and Extended Data Figs. 2–7 and 9 Statistical source data for Figs. 1–6 and Extended Data Figs. 2–7 and 9.

## Data Availability

Data are available in the Article or Supplementary Information. Source data are provided with this paper.
