# Supplementary material for: IL-12-releasing nanoparticles for effective immunotherapy of metastatic ovarian cancer
Source: Nat Mater. 2025 Oct 31;25(2):322–34. doi: 10.1038/s41563-025-02390-9 (PMC12867765; doi:10.1038/s41563-025-02390-9)
Supplement: Supplementary file 1 — Supplementary Figs. 1–8, Tables 1 and 2 and Note. [file 41563_2025_2390_MOESM1_ESM.pdf]

nature materials

Supplementary information https://doi.org/10.1038/s41563-025-02390-9

IL-12-releasing nanoparticles for effective immunotherapy of metastatic ovarian cancer

In the format provided by the authors and unedited

Supplementary Note

Although other approaches exist to augment cytokine delivery in cancer, most rely on local (intratumoral, i.t.) administration to increase tumor drug concentration while minimizing systemic exposure.[1] However, such approaches would be impractical to implement in metastatic OC due to difficulties with administration into small, disseminated legions in the i.p. space. Moreover, while NPs have previously been explored for cytokine delivery, they have primarily relied on passive tumor targeting through the enhanced permeability and retention (EPR) effect by extending NP circulatory half-life via polyethylene glycol (PEG) decoration.[2] However, in addition to concerns with anti-PEG immune responses[3], increased half-life also increases systemic exposure and subsequent toxicity, and the lack of specific tumor interactions would facilitate clearance from the peritoneal cavity after i.p. administration.[4] Notably, plasmid DNA complexed with lipids and polyethylenimine are under clinical investigation for peritoneal IL-12 expression post i.p. administration in OC patients.[5] While positive results have been obtained in a phase 2 trial[6], this therapy lacks any targeting mechanism, leading to a toxicity profile similar to prior clinical studies of i.p.-administered recombinant free IL-12[7–10]. Moreover, as it requires in vivo DNA delivery, there is a greater risk of patient-to-patient variability in the dose.

Current approaches to focus the action of IL-12 in tumors in the clinic have mainly focused on antibody-cytokine fusions.[11] However, unlike PLE-coated LbL NPs that resist endocytosis, antibodies are rapidly cleared from the extracellular milieu upon cell-surface antigen binding.[12] To overcome this limitation, systemically delivered IL-12-based immunocytokines mostly rely on binding to extracellular components such as necrotic DNA or fibronectin.[12] However, these strategies have been found to directly increase IL-12 toxicity[13] or increase systemic IFN-γ levels[14] with only modest (2-3 fold) increase in tumor exposure compared to controls. Moreover, antibodies have a comparatively short half-life in the i.p. space (<5 hrs in rodents).[15] Another approach in clinical development is the delivery of "masked" IL-12 that is activated by protease activity in the tumor microenvironment.[16] However, it is unclear if this approach will be effective in ovarian cancer, as many tumor proteases are also highly expressed in ascites fluid[17], limiting tumor specificity.

While the primary systemic administration route for many ovarian cancer drugs in the clinic is intravenous (i.v.), the LbL-NPs presented here were delivered i.p. based on the significantly improved tumor binding seen in our prior work with OC compared to i.v.[4,18] In

addition to a reduced trafficking barrier to reach the disseminated tumors in the peritoneum cavity (vasculature extravasation into the high-pressure ascites cavity), i.p. administration minimizes cytokine interaction with circulating immune cells which can both cause systemic toxicity and premature clearance of cytokine-based constructs.[19] In the context of OC, the i.p. administration is a standard-of-care route for chemotherapy treatment but has been less frequently used due to lack of benefit over i.v. with current chemotherapy regimens.[20,21] Nonetheless, it is a feasible administration route with various recently completed and ongoing clinical trials.[5,22–24]

References

1. Nguyen, K. G. *et al.* Localized Interleukin-12 for Cancer Immunotherapy. *Front Immunol* **11**, (2020).

2. Pires, I. S., Hammond, P. T. & Irvine, D. J. Engineering Strategies for Immunomodulatory Cytokine Therapies: Challenges and Clinical Progress. *Adv Ther (Weinh)* **4**, 2100035 (2021).

3. Chen, B.-M., Cheng, T.-L. & Roffler, S. R. Polyethylene Glycol Immunogenicity: Theoretical, Clinical, and Practical Aspects of Anti-Polyethylene Glycol Antibodies. *ACS Nano* **15**, 14022–14048 (2021).

4. Correa, S. *et al.* Tuning Nanoparticle Interactions with Ovarian Cancer through Layer-by-Layer Modification of Surface Chemistry. *ACS Nano* **14**, 2224–2237 (2020).

5. Thaker, P. H., Borys, N., Fewell, J. & Anwer, K. GEN-1 immunotherapy for the treatment of ovarian cancer. *Future Oncology* **15**, 421–438 (2019).

6. Fuerst, M. L. Boosting Overall Survival in Newly Diagnosed Advanced Ovarian Cancer. *Oncology Times* **46**, 31–31 (2024).

7. Lenzi, R. *et al.* Phase II study of intraperitoneal recombinant interleukin-12 (rhIL-12) in patients with peritoneal carcinomatosis (residual disease < 1 cm) associated with ovarian cancer or primary peritoneal carcinoma. *J Transl Med* **5**, 66 (2007).

8. Lenzi, R. *et al.* Phase I study of intraperitoneal recombinant human interleukin 12 in patients with Müllerian carcinoma, gastrointestinal primary malignancies, and mesothelioma. *Clin Cancer Res* **8**, 3686–95 (2002).

9. Thaker, P. H. *et al.* GEN-1 in Combination with Neoadjuvant Chemotherapy for Patients with Advanced Epithelial Ovarian Cancer: A Phase I Dose-escalation Study. *Clinical Cancer Research* **27**, 5536–5545 (2021).

10. Alvarez, R. D. *et al.* A phase II trial of intraperitoneal EGEN-001, an IL-12 plasmid formulated with PEG–PEI–cholesterol lipopolymer in the treatment of persistent or

recurrent epithelial ovarian, fallopian tube or primary peritoneal cancer: A Gynecologic Oncology Group study. *Gynecol Oncol* **133**, 433–438 (2014).

11. Pires, I. S., Hammond, P. T. & Irvine, D. J. Engineering Strategies for Immunomodulatory Cytokine Therapies: Challenges and Clinical Progress. *Adv Ther (Weinh)* **4**, 2100035 (2021).

12. Young, P. A., Morrison, S. L. & Timmerman, J. M. Antibody-Cytokine Fusion Proteins for Treatment of Cancer: Engineering Cytokines for Improved Efficacy and Safety. *Semin Oncol* **41**, 623–636 (2014).

13. Halin, C. *et al.* Enhancement of the antitumor activity of interleukin-12 by targeted delivery to neovasculature. *Nat Biotechnol* **20**, 264–269 (2002).

14. Fallon, J. *et al.* The immunocytokine NHS-IL12 as a potential cancer therapeutic. *Oncotarget* **5**, 1869–1884 (2014).

15. Barrett, J. S., Wagner, J. G., Fisher, S. J. & Wahl, R. L. Effect of intraperitoneal injection volume and antibody protein dose on the pharmacokinetics of intraperitoneally administered IgG2a kappa murine monoclonal antibody in the rat. *Cancer Res* **51**, 3434–44 (1991).

16. Patel, E. *et al.* XTX301, a Tumor-Activated Interleukin-12 Has the Potential to Widen the Therapeutic Index of IL12 Treatment for Solid Tumors as Evidenced by Preclinical Studies. *Mol Cancer Ther* **23**, 421–435 (2024).

17. Ghosh, S., Wu, Y. & Stack, M. S. Ovarian Cancer- Associated Proteinases. in 331–351 (2002). doi:10.1007/978-1-4757-3587-1_16.

18. Barberio, A. E. *et al.* Layer-by-layer interleukin-12 nanoparticles drive a safe and effective response in ovarian tumors. *Bioeng Transl Med* (2022) doi:10.1002/btm2.10453.

19. Tzeng, A., Kwan, B. H., Opel, C. F., Navaratna, T. & Wittrup, K. D. Antigen specificity can be irrelevant to immunocytokine efficacy and biodistribution. *Proceedings of the National Academy of Sciences* **112**, 3320–3325 (2015).

20. Monk, B. J. & Chan, J. K. Is intraperitoneal chemotherapy still an acceptable option in primary adjuvant chemotherapy for advanced ovarian cancer? *Annals of Oncology* **28**, viii40–viii45 (2017).

21. Markman, M. & Walker, J. L. Intraperitoneal Chemotherapy of Ovarian Cancer: A Review, With a Focus on Practical Aspects of Treatment. *Journal of Clinical Oncology* **24**, 988–994 (2006).

22. Knisely, A. *et al.* Phase 1b study of intraperitoneal ipilimumab and nivolumab in patients with recurrent gynecologic malignancies with peritoneal carcinomatosis. *Med* **5**, 311-320.e3 (2024).

23. Cutri-French, C., Nasioudis, D., George, E. & Tanyi, J. L. CAR-T Cell Therapy in Ovarian Cancer: Where Are We Now? *Diagnostics* **14**, 819 (2024).

24. Corbaux, P. *et al.* Intraperitoneal Nivolumab after Debulking Surgery and Hyperthermic Intraperitoneal Chemotherapy in Advanced Ovarian Cancer: A Phase I Study with Expansion Cohort. *Clinical Cancer Research* **30**, 3438–3446 (2024).

Supplementary Table 1: Lipid composition of nanoparticles and summary characteristics.

Lipid or trait	Nickel-headgroup	Maleimide-headgroup
DSPC; 1,2-distearoyl-sn-glycero-3-phosphocholine	65 mol%	65 mol%
Cholesterol	23.9 mol%	23.9 mol%
POPG; 1-palmitoyl-2-oleoyl-sn-glycero-3-phospho-(1'-rac-glycerol) (sodium salt)	6.1 mol%	6.1 mol%
18:1 DGS-NTA(Ni); 1,2-dioleoyl-sn-glycero-3-[(N-(5-amino-1-carboxypentyl)iminodiacetic acid)succinyl] (nickel salt)	5 mol%	0 mol%
18:1 MPB-PE; 1,2-dioleoyl-sn-glycero-3-phosphoethanolamine-N-[4-(p-maleimidophenyl)butyramide] (sodium salt)	0 mol%	5 mol%
Diameter (Z-avg) unlayered (UL) ± S.D.	86.3 ± 4.2 nm	87.7 ± 4.0 nm
Diameter (#-avg) unlayered (UL) ± S.D.	60.2 ± 5.3 nm	57.8 ± 2.2 nm
PDI unlayered (UL) ± S.D.	0.08 ± 0.04	0.14 ± 0.05
Zeta potential unlayered (UL) ± S.D.	-58 ± 11 mV	-63 ± 11 mV
Diameter (Z-avg) PLR-PLE (LbL) ± S.D.	121.3 ± 4.5 nm	122.5 ± 2.5 nm
Diameter (#-avg) PLR-PLE (LbL) ± S.D.	84.7 ± 6.6 nm	81.2 ± 1.1 nm
Zeta potential PLR-PLE (LbL) ± S.D.	-63 ± 4 mV	-63 ± 1 mV
PDI PLR-PLE (LbL) ± S.D.	0.11 ± 0.05	0.13 ± 0.02

Supplementary Table 2: Lipid composition of SAT NPs.

Lipid	SAT NPs (mol%)
DSPC; 1,2-distearoyl-sn-glycero-3-phosphocholine	65%
Cholesterol	23.9%
DSPG; 1,2-distearoyl-sn-glycero-3-phospho-(1'-rac-glycerol) (sodium salt)	6.1%
16:0 MPB-PE; 1,2-dipalmitoyl-sn-glycero-3-phosphoethanolamine-N-[4-(p-maleimidophenyl)butyramide] (sodium salt)	5%

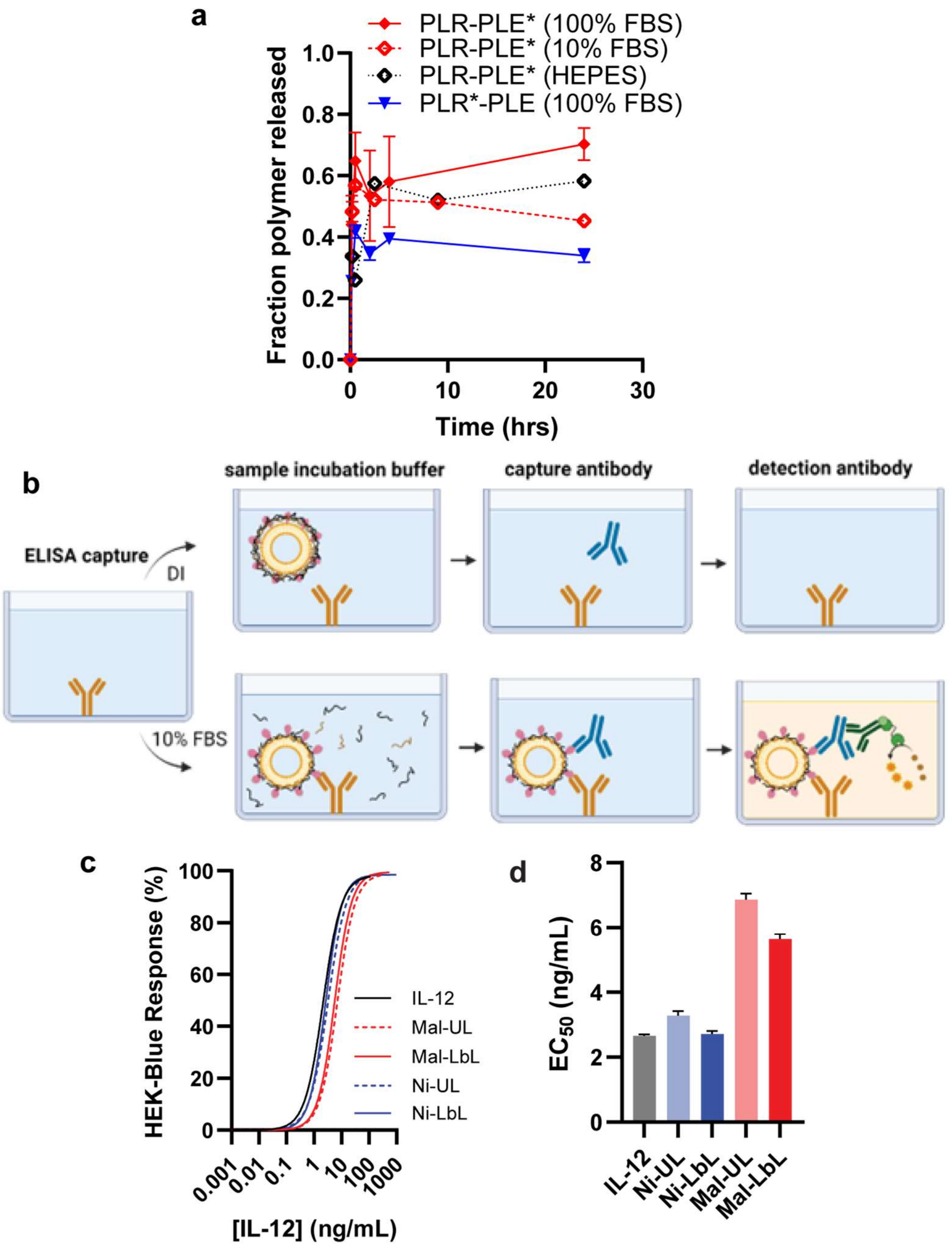

Supplementary Figure 1. Polyelectrolyte films are partially released in buffers with physiological ionic strength and does not block IL-12 availability in LbL-NPs. A, Measurement of PLE or PLR release from LbL film on NPs incubated at 37 °C in 15 mM HEPES 150 mM NaCl (HEPES), 10%, or 100% FBS (mean ± s.d.). **b,** Schematic for monoclonal antibody capture of NP-bound IL-12 and detection in varying buffer conditions. **c,** HEK-Blue IL-12 reporter cell line response to IL-12 in various formats (*n*>100 points per curve from 7 independent particle batches). (**d**) Calculated IL-12 EC_{50} from HEK-Blue IL12 response curves (mean ± s.e.m).

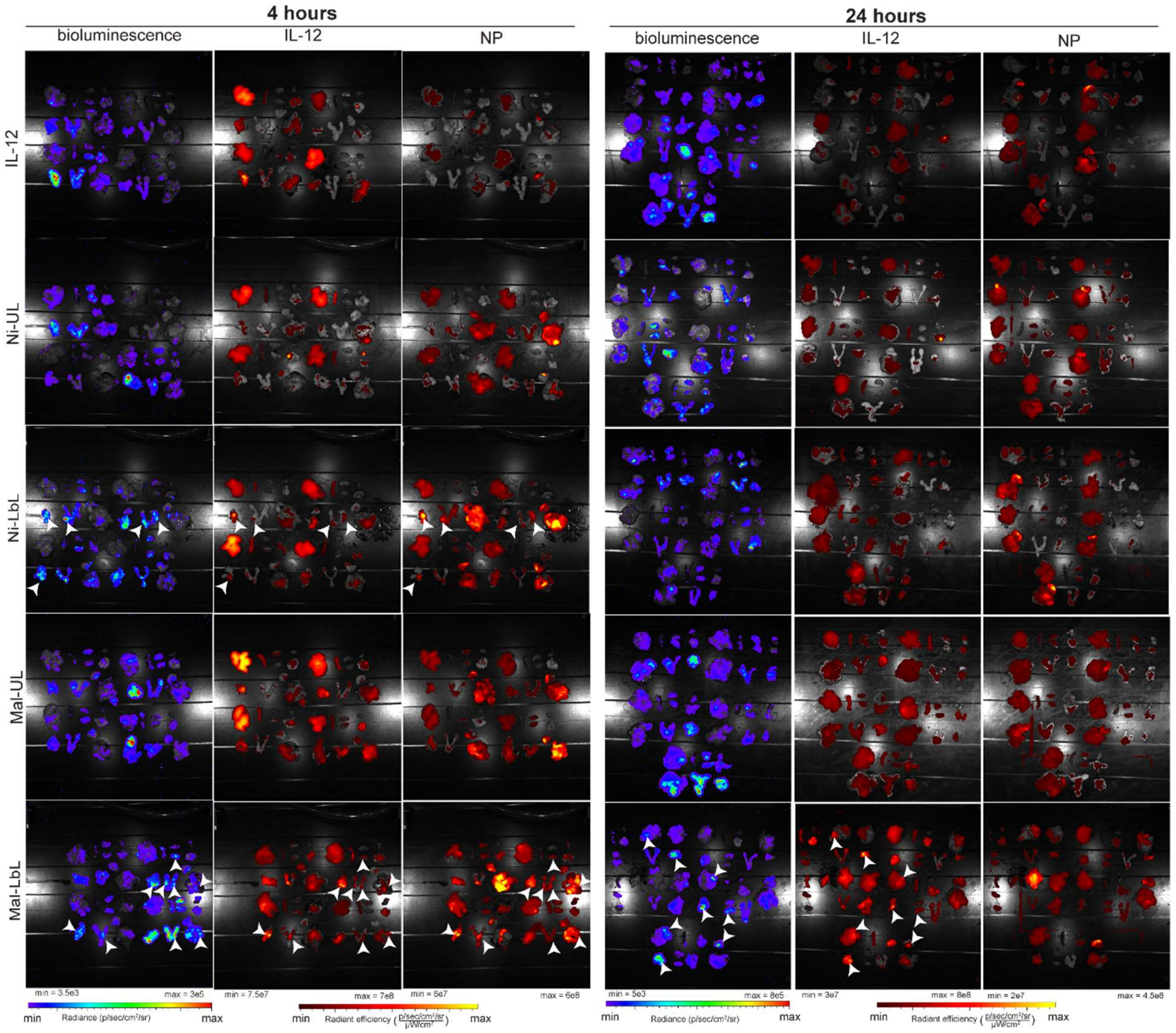

Supplementary Figure 2. Ex-vivo IVIS organ images. B6C3F1 mice (n=5/group) inoculated with 10^6 HM-1-luc tumor cells on day 0 were administered fluorescently-tagged NPs carrying 20 μg of fluorescently tagged IL-12 on day 14. Four hours or one day after dosing, animals were sacrificed, and tissues were analyzed ex-vivo via IVIS. Arrows indicate colocalization of the tumor BLI and IL-12 or NP.

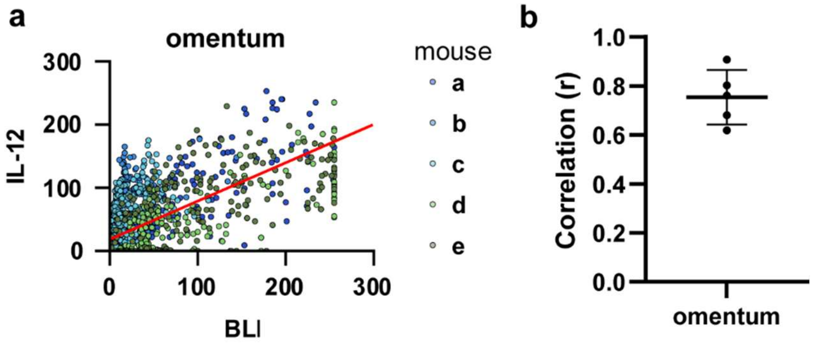

Supplementary Figure 3. Pixel-by-pixel correlation of ex-vivo omentum tissue 24 hrs after fluorescent IL-12 dosing. a-c, B6C3F1 mice (n=5) inoculated with 10^6 HM-1-luc tumor cells on day 0 were administered fluorescently-tagged Mal-LbL NPs carrying 20 µg of fluorescently tagged IL-12 on day 14. One day after dosing, animals were sacrificed, and tissues were analyzed ex-vivo via IVIS**.** Shown are pixel intensity values of IL-12 fluorescence and BLI that were extracted from IVIS images of omentum tissue from Mal-LbL treated mice (**a**), and the resulting derived Spearman's Correlation (**b**).

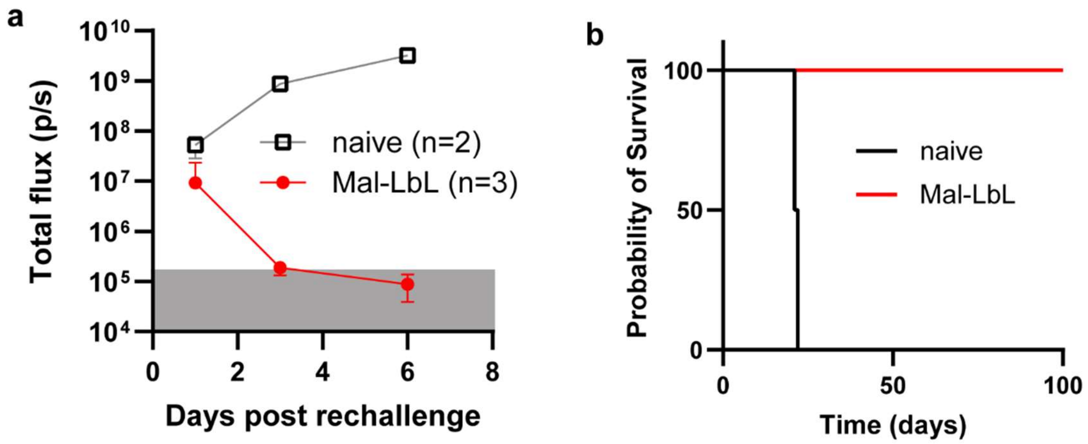

Supplementary Figure 4. Mice with complete remission of metastatic ovarian cancer demonstrate strong immune memory induction upon i.p. luc-HM-1 rechallenge. a-b, B6C3F1 mice (n = 10/group) inoculated with 10^6 HM-1-luc tumor cells on day 0 were treated on days 7 and 14 with 20 µg of IL-12 as a free cytokine or conjugated to NPs. On day 100, surviving Mal-LbL mine (n = 3) or naïve (n = 2) were injected with $3x10^5$ luc-HM-1 cells i.p. Shown are in vivo IVIS whole-animal i.p. BLI readings (mean ± s.e.m., **b**), and overall survival (**c**).

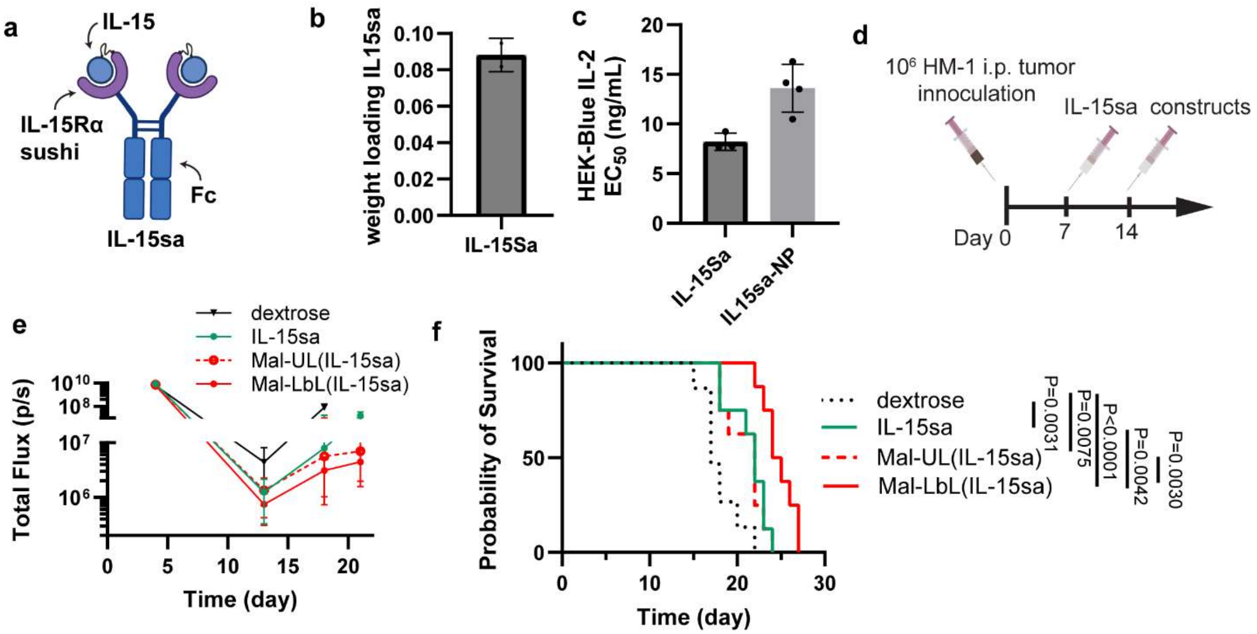

Supplementary Figure 5. IL-15sa loaded Mal-LbL NPs outperform free cytokine a, schematic of IL-15sa. **b,** weight loading of IL-15sa onto Mal-LbL NPs (mean ± s.d., each point represents the average triplicate of independent batches). **c**, Derived HEK-Blue IL-2 reporter cell line EC_{50} to IL-15sa as free cytokine or in NP format (mean ± s.d.), . **d-f,** B6C3F1 mice (1 cohort *n* = 8/group) inoculated with 10^6 HM-1-luc tumor cells on day 0 were treated on days 7 and 14 with 10 µg of IL-15sa as a free cytokine or conjugated to NPs. Shown are the experimental timeline **(d),** in vivo IVIS whole-animal i.p. BLI readings (mean ± s.d., **e)**, and overall survival **(f)**. Statistical comparisons between survival curves were performed using a log-rank (Mantel–Cox) test.

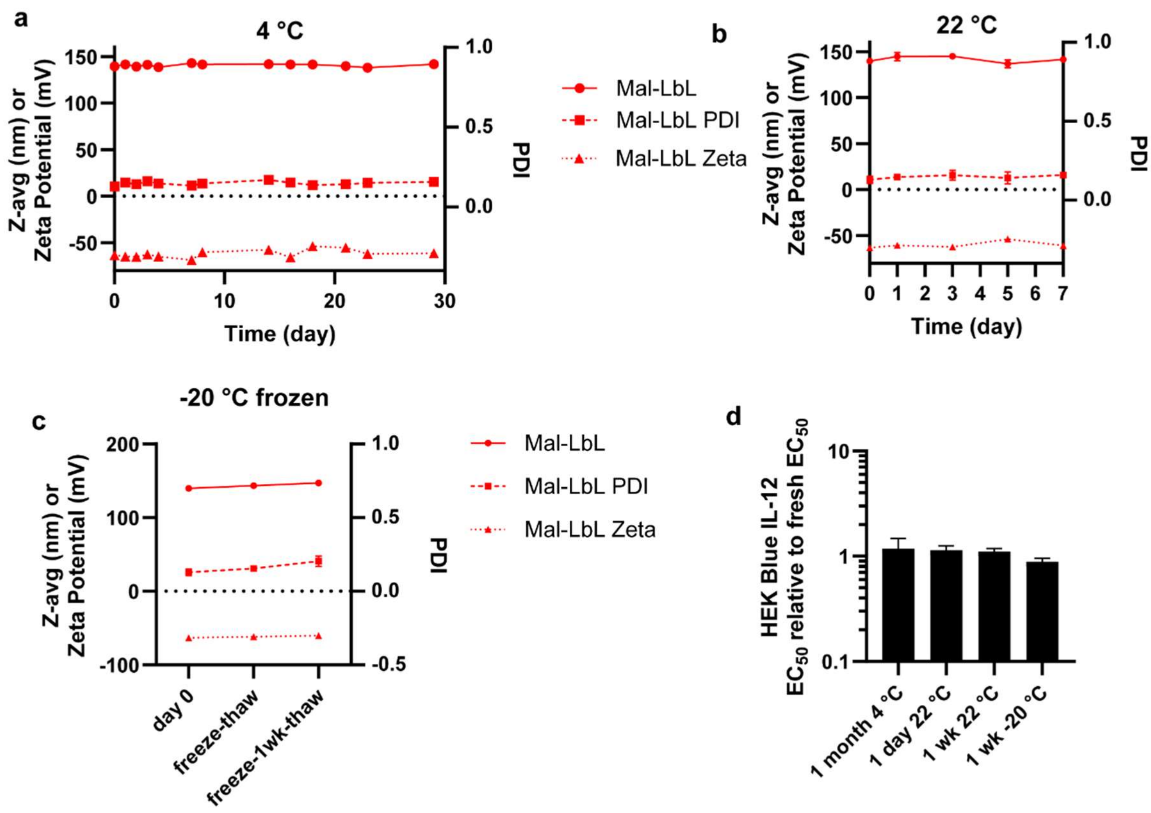

Supplementary Figure 6. Mal-LbL NPs conjugated with IL-12 maintain colloidal stability and IL-12 bioactivity under storage. a-c, Hydrodynamic size (Z-avg), PDI, and zeta potential of Mal-LbL NPs stored at 4 °C (mean ± s.d., a), 22 °C (mean ± s.d., b), or frozen at -20 °C (mean ± s.d., c). d, derived HEK-Blue IL-12 reporter cell line EC50 to IL-12 in Mal-LbL NPs after various storage conditions and times (mean ± 95% CI).

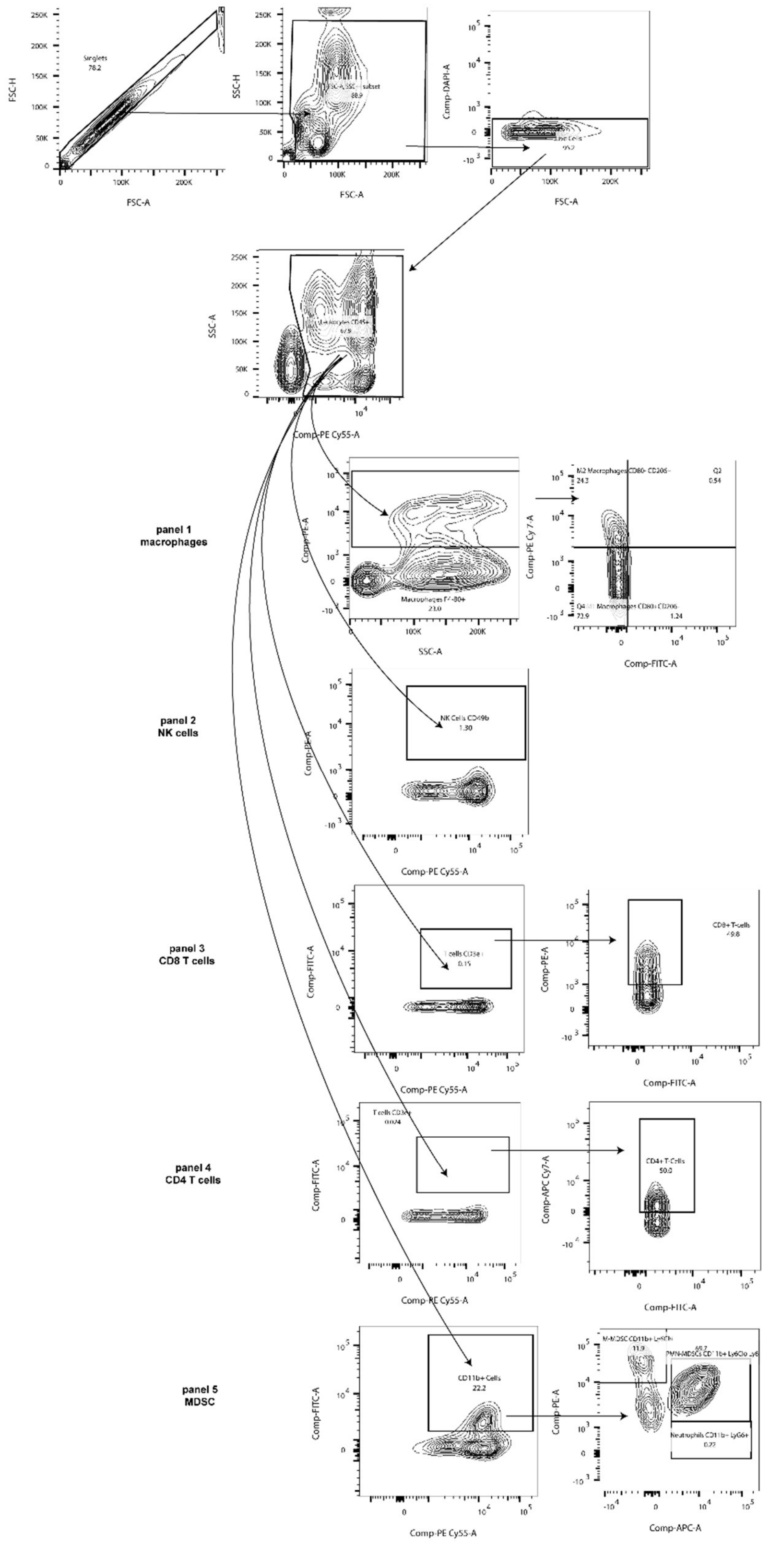

Supplementary Figure 7. Flow cytometry cell gating strategy for general immune phenotyping.

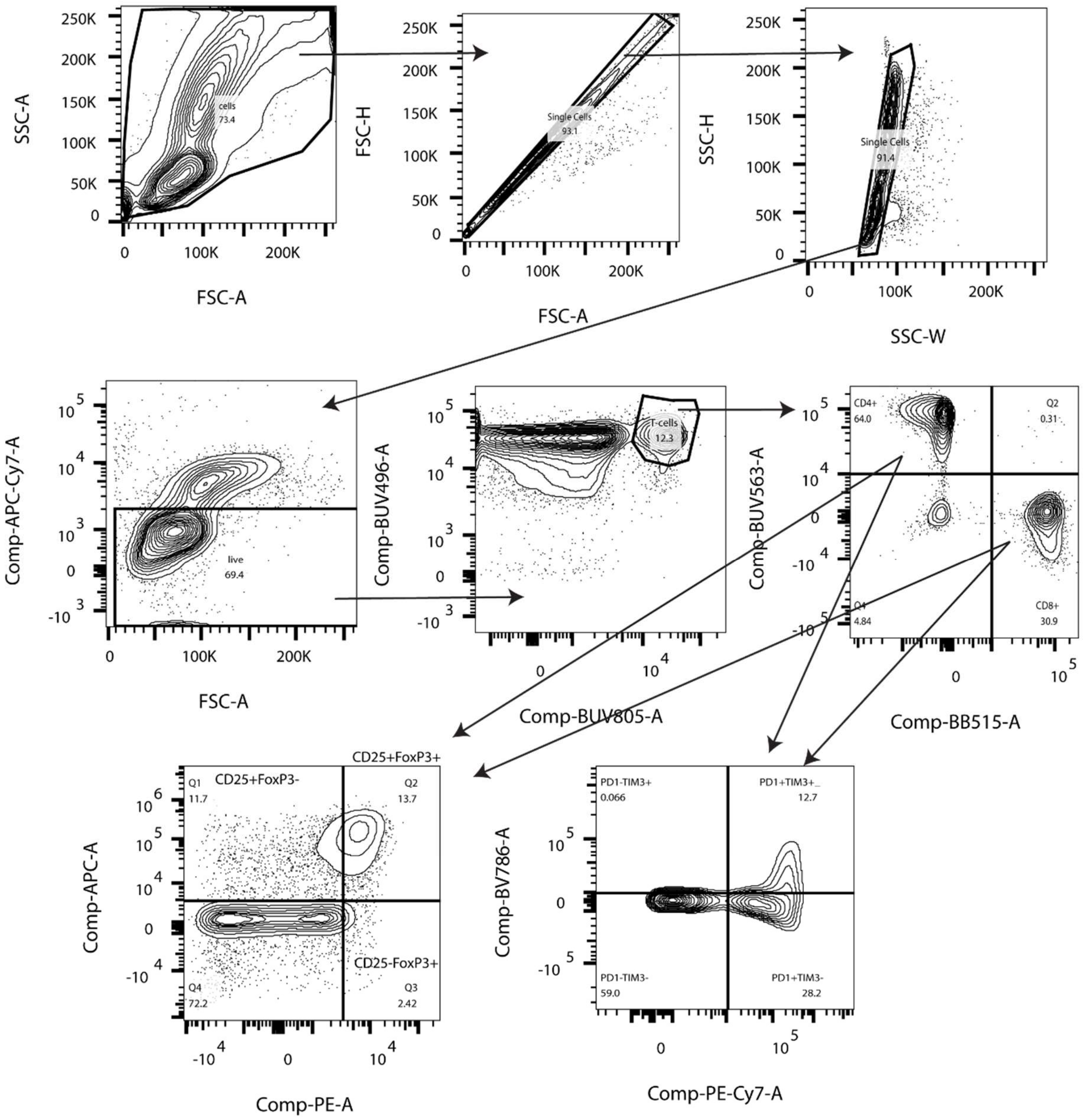

Supplementary Figure 8. Flow cytometry cell gating strategy for T cell characterization.
