# Supplementary material for: IL-12-releasing nanoparticles for effective immunotherapy of metastatic ovarian cancer
Source: Nat Mater. 2025 Oct 31;25(2):322–34. doi: 10.1038/s41563-025-02390-9 (PMC12867765; doi:10.1038/s41563-025-02390-9)
Supplement: Supplementary file 2 — Reporting Summary [file 41563_2025_2390_MOESM2_ESM.pdf]

nature portfolio

Corresponding author(s): Darrell J. Irvine and Paula T. Hammond

Last updated by author(s): 2025 08 09

Reporting Summary

Nature Portfolio wishes to improve the reproducibility of the work that we publish. This form provides structure for consistency and transparency in reporting. For further information on Nature Portfolio policies, see our Editorial Policies and the Editorial Policy Checklist.

Statistics

For all statistical analyses, confirm that the following items are present in the figure legend, table legend, main text, or Methods section.

n/a	Confirmed	
☐	☒	The exact sample size (*n*) for each experimental group/condition, given as a discrete number and unit of measurement
☐	☒	A statement on whether measurements were taken from distinct samples or whether the same sample was measured repeatedly
☐	☒	The statistical test(s) used AND whether they are one- or two-sided<br>*Only common tests should be described solely by name; describe more complex techniques in the Methods section.*
☒	☐	A description of all covariates tested
☐	☒	A description of any assumptions or corrections, such as tests of normality and adjustment for multiple comparisons
☐	☒	A full description of the statistical parameters including central tendency (e.g. means) or other basic estimates (e.g. regression coefficient) AND variation (e.g. standard deviation) or associated estimates of uncertainty (e.g. confidence intervals)
☐	☒	For null hypothesis testing, the test statistic (e.g. *F*, *t*, *r*) with confidence intervals, effect sizes, degrees of freedom and *P* value noted<br>*Give P values as exact values whenever suitable.*
☒	☐	For Bayesian analysis, information on the choice of priors and Markov chain Monte Carlo settings
☒	☐	For hierarchical and complex designs, identification of the appropriate level for tests and full reporting of outcomes
☒	☐	Estimates of effect sizes (e.g. Cohen's *d*, Pearson's *r*), indicating how they were calculated

Our web collection on statistics for biologists contains articles on many of the points above.

Software and code

Policy information about availability of computer code

Data collection	Particle size and zeta potential measurements were measured on a Zetasizer Nano ZSP and associated software. Nanoparticle micrographs were acquired using Transmission Electron Microscopy (TEM) on a JEOL 2100F microscope and accompanying software. Plate-based fluorescent/absorbance data were measured using the Tecan Infinite M200 Pro plate reader and accompanying software. Confocal images were taken on an Olympus FV1200 laser scanning confocal microscope and associated software. Flow cytometry data were obtained using BD FACSDiva software. FACS data were obtained using a BD LSR Fortessa and accompanying software.
Data analysis	FlowJo 10.8.2 was used for the analysis of flow cytometry data. GraphPad Prism 10.1 was used for plotting and statistical analysis. Microsoft Excel (v16.8) was used to organize data. Microsoft Word (v16.8), and Adobe Illustrator (v27.7) were used to draft the manuscript.

For manuscripts utilizing custom algorithms or software that are central to the research but not yet described in published literature, software must be made available to editors and reviewers. We strongly encourage code deposition in a community repository (e.g. GitHub). See the Nature Portfolio guidelines for submitting code & software for further information.

Data

Policy information about availability of data

All manuscripts must include a data availability statement. This statement should provide the following information, where applicable:

- Accession codes, unique identifiers, or web links for publicly available datasets
- A description of any restrictions on data availability
- For clinical datasets or third party data, please ensure that the statement adheres to our policy

Data supporting the findings of this study are available within the paper and its supplementary information. Any data supporting the results of this study are also available from corresponding authors on reasonable request. Source data are provided with this paper.

Research involving human participants, their data, or biological material

Policy information about studies with human participants or human data. See also policy information about sex, gender (identity/presentation), and sexual orientation and race, ethnicity and racism.

Reporting on sex and gender	This study did not involve human research participants.
Reporting on race, ethnicity, or other socially relevant groupings	*Please specify the socially constructed or socially relevant categorization variable(s) used in your manuscript and explain why they were used. Please note that such variables should not be used as proxies for other socially constructed/relevant variables (for example, race or ethnicity should not be used as a proxy for socioeconomic status).*<br>*Provide clear definitions of the relevant terms used, how they were provided (by the participants/respondents, the researchers, or third parties), and the method(s) used to classify people into the different categories (e.g. self-report, census or administrative data, social media data, etc.)*<br>*Please provide details about how you controlled for confounding variables in your analyses.*
Population characteristics	*Describe the covariate-relevant population characteristics of the human research participants (e.g. age, genotypic information, past and current diagnosis and treatment categories). If you filled out the behavioural & social sciences study design questions and have nothing to add here, write "See above."*
Recruitment	*Describe how participants were recruited. Outline any potential self-selection bias or other biases that may be present and how these are likely to impact results.*
Ethics oversight	*Identify the organization(s) that approved the study protocol.*

Note that full information on the approval of the study protocol must also be provided in the manuscript.

Field-specific reporting

Please select the one below that is the best fit for your research. If you are not sure, read the appropriate sections before making your selection.

☒ Life sciences ☐ Behavioural & social sciences ☐ Ecological, evolutionary & environmental sciences

For a reference copy of the document with all sections, see nature.com/documents/nr-reporting-summary-flat.pdf

Life sciences study design

All studies must disclose on these points even when the disclosure is negative.

Sample size	sample sizes were determined to obtain statistical significance on the basis of preliminary studies and of prior work from our laboratory in similar experiments.
Data exclusions	No data were excluded.
Replication	Experiments were repeated to confirm reproducibility, with specific sample sizes and details indicated in figure captions.
Randomization	For all tumor studies, animals were randomized into groups with comparable tumor burdens.
Blinding	No blinding was done for this study.

Reporting for specific materials, systems and methods

We require information from authors about some types of materials, experimental systems and methods used in many studies. Here, indicate whether each material, system or method listed is relevant to your study. If you are not sure if a list item applies to your research, read the appropriate section before selecting a response.

Materials & experimental systems

n/a	Involved in the study
☐	☒ Antibodies
☐	☒ Eukaryotic cell lines
☒	☐ Palaeontology and archaeology
☐	☒ Animals and other organisms
☒	☐ Clinical data
☒	☐ Dual use research of concern
☒	☐ Plants

Methods

n/a	Involved in the study
☒	☐ ChIP-seq
☐	☒ Flow cytometry
☒	☐ MRI-based neuroimaging

Antibodies

Antibodies used

In vivo immune checkpoint inhibitors: anti-PD1 antibody (clone 29F.1A12, BioXCell) and anti-CLTA4 antibody (clone 9D9, BioXCell)

For immunophenotyping (all mouse targets), see antibodies table below:

Target	Clone	Fluorophore	Source	Dilution
CD3ε	145-2C11	FITC	BD Biosciences	1:100
CD3ε	145-2C11	BUV805	BD Biosciences	1:100
CD49b	DX5	PE	BD Biosciences	1:100
CD45	30-F11	PE-Cy5.5	BD Biosciences	1:100
CD45	30-F11	BUV496	BD Biosciences	1:100
F4/80	T45-2342	PE	BD Biosciences	1:100
CD4	GK1.5	BUV563	BD Biosciences	1:100
CD80	16-10A1	FITC	BD Biosciences	1:100
CD11b	M1/70	FITC	BD Biosciences	1:100
Ly6C	AL-21	PE	BD Biosciences	1:100
CD4	GK1.5	APC-Cy7	BioLegend	1:100
CD8α	53-6.7	PE	BioLegend	1:100
CD206	C068C2	PE-Cy5	BioLegend	1:100
CD279 (PD-1)	29F.1A12	PE-Cy7	BioLegend	1:100
CD366 (Tim-3)	RMT3-23	BV785	BioLegend	1:100
CD25	PC61	BV421	BioLegend	1:100
Ly6G	1A8	APC	BioLegend	1:100
CD8α	KT15	FITC	Invitrogen	1:100
FoxP3	FJK-16s	PE	Invitrogen	1:100

Validation

All antibodies were validated by the vendors: Biolegend, BD Biosciences, and BioXCell.

Eukaryotic cell lines

Policy information about cell lines and Sex and Gender in Research

Cell line source(s)

OV2944-HM1 cells were obtained from RIKEN BRC cell bank. HM-1 cells expressing luciferase (HM-1-luc) were derived as as described in a previous publication (Toyoshima et al. Luminescence. 2009 24(5):324-31).

HEK-Blue IL-12 cells were purchased from Invivogen.

Authentication

Each cell line was maintained separately and stocked in early passages to minimize contamination and to preserve cell identity.

Mycoplasma contamination

The cell lines were periodically tested and confirmed to be free of mycoplasma contamination.

Commonly misidentified lines
(See ICLAC register)

No commonly misidentified cell lines were used.

Animals and other research organisms

Policy information about studies involving animals; ARRIVE guidelines recommended for reporting animal research, and Sex and Gender in Research

Laboratory animals

Female B6C3F1 mice were all obtained from Jackson Laboratories. Mice were between 8-12 weeks old at the start of all studies and weighed approximately 18-20 g.

Wild animals

The study did not involve wild animals.

Reporting on sex | All studies employed female mice for evaluation of ovarian cancer models.

Field-collected samples | The study did not involve samples collected from the field.

Ethics oversight | All animal studies and procedures were carried out following federal, state and local guidelines under an animal protocol approved by the institutional animal care and use committee at MIT (protocol numbers: 2303000488 and 082105224)

Note that full information on the approval of the study protocol must also be provided in the manuscript.

Plants

Seed stocks | *Report on the source of all seed stocks or other plant material used. If applicable, state the seed stock centre and catalogue number. If plant specimens were collected from the field, describe the collection location, date and sampling procedures.*

Novel plant genotypes | *Describe the methods by which all novel plant genotypes were produced. This includes those generated by transgenic approaches, gene editing, chemical/radiation-based mutagenesis and hybridization. For transgenic lines, describe the transformation method, the number of independent lines analyzed and the generation upon which experiments were performed. For gene-edited lines, describe the editor used, the endogenous sequence targeted for editing, the targeting guide RNA sequence (if applicable) and how the editor was applied.*

Authentication | *Describe any authentication procedures for each seed stock used or novel genotype generated. Describe any experiments used to assess the effect of a mutation and, where applicable, how potential secondary effects (e.g. second site T-DNA insertions, mosiacism, off-target gene editing) were examined.*

Flow Cytometry

Plots

Confirm that:

☒ The axis labels state the marker and fluorochrome used (e.g. CD4-FITC).

☒ The axis scales are clearly visible. Include numbers along axes only for bottom left plot of group (a 'group' is an analysis of identical markers).

☒ All plots are contour plots with outliers or pseudocolor plots.

☒ A numerical value for number of cells or percentage (with statistics) is provided.

Methodology

Sample preparation | Two days after dosing, mice were bled retro-orbitally and then euthanized to extract ascites cells via peritoneal lavage with PBS. Peritoneal tumor nodules and spleen were also collected. Part of the blood samples were submitted to The Division of Comparative Medicine at MIT to perform a complete blood count and analysis of liver function and the remainder was processed with ACK lysing buffer (Gibco) to isolate PBMCs. Spleens were processed on a 70 µm cell strainer with a syringe plunger then exposed to ACK lysing buffer to lyse red blood cells (RBCs). Tumor nodules from each mouse were diced with scissors then incubated for one hour at 37 °C in 2 mL of 1 mg/mL collagenase type IV (Sigma) in RPMI media. After collagenase incubation, tumors were processed on a 70 µm cell strainer then collected with an insulin syringe into falcon tubes to pellet tumor cells and wash out collagenase solution. For cell staining with antibodies, samples were placed in 96 well plates, then centrifuged and resuspended in Fc block solution (BD Biosciences) for 5 minutes. Freshly prepared antibody panels were then mixed with the samples and allowed to react for 20 minutes. Finally, DAPI (BD Biosciences) was added to each well (at 2 µg/mL) and allowed to react for 5 minutes. Stained cells were washed twice with flow cytometry buffer (PBS with 0.5% BSA and 2 mM EDTA), then resuspended in 2% PFA in PBS for 30 minutes, washed and stored at 4 °C for analysis the next day on a on a flow cytometry instrument (LSR Fortessa, BD Biosciences). Flow cytometry buffer was used to prepare Fc block and antibody solutions.

Instrument | Cells were analysed using BD FACS LSR Fortessa.

Software | BD FACSDiva was used for the collection of FACS data and FlowJo was used for data analysis. The collected data were plotted with statistical analysis by GraphPad Prism.

Cell population abundance | No cell sorting was performed.

Gating strategy | Example gating strategies are provided throughout in the Supplementary information.

☒ Tick this box to confirm that a figure exemplifying the gating strategy is provided in the Supplementary Information.
